# Associations of reproductive factors with incidence of myocardial infarction and ischemic stroke in postmenopausal women: a cohort study

**DOI:** 10.1186/s12916-023-02757-2

**Published:** 2023-02-20

**Authors:** Su-Min Jeong, Jung Eun Yoo, Keun Hye Jeon, Kyungdo Han, Heesun Lee, Dong-Yun Lee, Dong Wook Shin

**Affiliations:** 1 Department of Medicine, Seoul National University College of Medicine, Seoul, Republic of Korea; 2 Department of Family Medicine, Seoul National University Health Service Center, Seoul, Republic of Korea; 3 Department of Family Medicine, Seoul National University Hospital, Seoul, Republic of Korea; 4 Department of Family Medicine, Healthcare System Gangnam Center, Seoul National University Hospital, Seoul, Republic of Korea; 5 Department of Family Medicine, CHA Gumi Medical Center, CHA University, Gumi, Republic of Korea; 6 Department of Statistics and Actuarial Science, Soongsil University, Seoul, Republic of Korea; 7 Department of Cardiology, Healthcare System Gangnam Center, Seoul National University Hospital, Seoul National University College of Medicine, Seoul, Korea; 8 Department of Obstetrics and Gynecology, Samsung Medical Center, Sungkyunkwan University School of Medicine, Seoul, Republic of Korea; 9 Department of Family Medicine/Supportive Care Center, Samsung Medical Center, Seoul, Republic of Korea; 10 Department of Clinical Research Design & Evaluation, Samsung Advanced Institute for Health Science and Technology (SAIHST), Sungkyunkwan University, 81 Irwon-Ro, Gangnam-Gu, Seoul, 06351 Republic of Korea

**Keywords:** Postmenopausal women, Cardiovascular disease, Age at menarche, Age at menopause, Reproductive span

## Abstract

**Background:**

To assess the association between the reproductive factors of age at menarche, age at menopause, and reproductive span and the incidence of myocardial infarction (MI) and ischemic stroke (IS).

**Methods:**

We used a population-based retrospective cohort study from the National Health Insurance Service database of Korea including a total of 1,224,547 postmenopausal women. Associations between age at menarche (≤ 12, 13–14 [reference], 15, 16, and ≥ 17 years), age at menopause (< 40, 40–45, 46–50, 51–54 [reference], and ≥ 55 years), and reproductive span (< 30, 30–33, 34–36, 37–40 [reference], and ≥ 41 years) and the incidence of MI and IS were assessed by Cox proportional hazard models with adjustment for traditional cardiovascular risk factors and various reproductive factors.

**Results:**

During a median follow-up of 8.4 years, 25,181 MI and 38,996 IS cases were identified. Late menarche (≥ 16 years), early menopause (≤ 50 years), and short reproductive span (≤ 36 years) were linearly associated with a 6%, 12–40%, and 12–32% higher risk of MI, respectively. Meanwhile, a U-shaped association between age at menarche and risk of IS was found, with a 16% higher risk in early menarche (≤ 12 years) and a 7–9% higher risk in late menarche (≥ 16 years). Short reproductive span was linearly associated with an increased risk of MI, whereas both shorter and longer reproductive spans were associated with an increased risk of IS.

**Conclusions:**

This study demonstrated different patterns of association between age at menarche and incidence of MI and IS: a linear association for MI versus a U-shaped association for IS. Female reproductive factors in addition to traditional cardiovascular risk factors should be considered when assessing overall cardiovascular risk in postmenopausal women.

**Supplementary Information:**

The online version contains supplementary material available at 10.1186/s12916-023-02757-2.

## Background

The burden of cardiovascular disease (CVD) continues to increase globally with 523 million cases and 18.6 million deaths in 2019 [1]. CVD is the leading cause of death for women accounting for 35% of total deaths in women in 2019 [2]. Among women, the notable increase in coronary heart disease risk after menopause has led to the hypothesis that alterations in levels of endogenous sex hormones, especially estrogen, could contribute to chronological changes in CVD risk [3]. There is abundant evidence that estrogen protects against atherosclerosis by promoting vasodilation, reducing fibrosis, and improving mitochondrial function and anti-oxidant activity [4].

Puberty and menopause transition are periods with remarkable changes in hormones, especially estradiol secretion. The timing of menarche and menopause varies depending on several factors, including estrogen levels, around these two turning points [5]. In this context, the relationships between reproductive factors, such as age at menopause [6–11] and age at menarche [6–8, 10, 12–15], with risk of coronary heart disease (CHD) and stroke have been evaluated in several studies (Additional file 1: Table S1). Early menarche is generally associated with an increased risk of CHD and stroke [7, 8, 13, 16], while some studies have reported an increased risk with late menarche [10], a U-shaped association [14, 15], or a null association [12]. Furthermore, there is a possibility that such association may differ according to CVD types. For example, a recent umbrella review reported that early menopause was associated with a 1.5-fold higher risk of ischemic heart disease, but no association was found with stroke [17]. Early menarche (< 12 years) was associated with a 1.2-fold higher risk of stroke, but not with CHD (adjusted hazard ratio [aHR] 1.08, 95% confidence interval [CI] 0.97–1.20) [6].

Reproductive span is a simple way to estimate the lifelong period with endogenous estrogen exposure, calculated by the difference between the timing of menarche and menopause. The association between reproductive span and CVD incidence has been investigated, but mostly in Western populations with conflicting results [18, 19]. A shorter reproductive span is generally associated with a higher risk of CVD events [18]. In a recent pooled analysis from 12 cohort studies, women with a short reproductive span (< 30 years) had a 71% higher risk of CVD [19], both for CHD and stroke. Similar results have been reported in studies of Asian women [10, 12]. However, a study in Korea reported a U-shaped association between reproductive span and incidence of CHD, while a short reproductive span was associated with a higher risk of stroke [10]. In addition, there might be interactions between reproductive factors (e.g., age at menarche and reproductive span) on the incidence of CVD. A previous study suggested that the timing of menarche affected the association between reproductive life span and incidence of CVD events, showing that early menarche (age ≤ 12 years) strengthened the association between short reproductive span and higher risk of CVD [19]. To our knowledge, such associations have not been investigated in an Asian female population.

Therefore, our aim in this study was to evaluate the associations between age at menarche, age at menopause, and reproductive span and incidence of CVD (myocardial infarction [MI] and ischemic stroke) among postmenopausal women using a large Korean population-based database. In addition, we investigated the combined effects of reproductive factors on the incidence of MI and ischemic stroke.

## Methods

### Data source and study setting

The present study used data from the National Health Insurance Services (NHIS) database of Korea. The KNHIS is a universal health coverage system that provides mandatory universal comprehensive medical care to 97% of the Korean population and an additional medical aid program to the 3% of the population in the lowest income bracket.

The NHIS database contains demographic information (e.g., age, sex, and income) in addition to claim information (general information on specifications, consultation statements, diagnosis statements defined by the International Classification of Disease 10th revision (ICD-10), and prescription statements) [20, 21].

Furthermore, because NHIS recommends free biennial cardiovascular health screening for all Koreans, health screening information (self-questionnaire on health behavior [e.g., past medical history, smoking, and drinking], anthropometric measurements [e.g., body mass index (BMI) and blood pressure], and laboratory test results [e.g., fasting glucose and lipid levels]) is also available for a large proportion of the population [22]. In addition, the National Cancer Screening Program (NCSP) was introduced in 1999, including breast and cervical cancer screening for women [23]. As a part of the NCSP, all Korean women over 40 years are screened for breast cancer biennially, and participants are asked to complete a questionnaire on reproductive factors. This nationwide database is widely used in epidemiological studies [24], including the studies on reproductive factors and various health outcomes [25].

### Study population

**Fig. 1:**
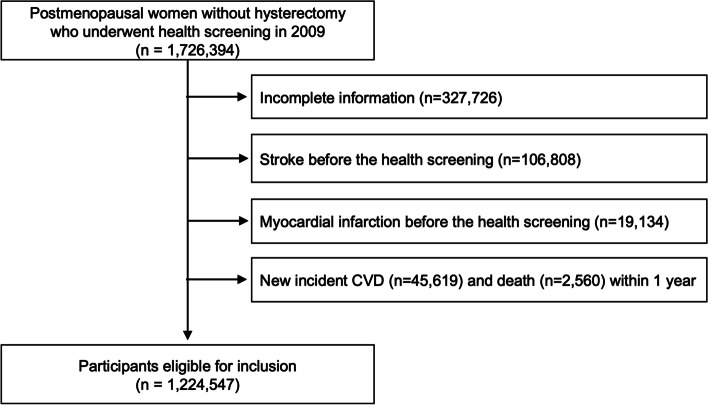
Flow chart of the study population

We initially identified 1,726,394 postmenopausal women without hysterectomy who underwent both cardiovascular and breast/cervical cancer screening in 2009. We first excluded 327,726 participants with missing data for at least one variable for complete case analysis (Additional file 1: Table S2). We also excluded participants who had a previous diagnosis of stroke (*n* = 106,808) or MI (*n* = 19,134). Participants who had a new CVD diagnosis (*n* = 45,619) or died (*n* = 2560) within 1 year after the health screening date were also excluded. A total of 1,224,547 postmenopausal women were included in the final analyses (Fig. 1).

This study was approved by the Institutional Review Board of Samsung Medical Center (IRB File No. SMC 2019–07-045). The review board waived the requirement for written informed consent because publicly open and anonymous data were analyzed in a retrospective manner.

### Reproductive factors

According to the NCSP guidelines, participants of cervical and breast cancer screening completed a questionnaire addressing their age at menarche and age at menopause in the form of short answers. Reproductive span was estimated as the difference between age at menarche and age at menopause [25, 26]. Considering the distribution of age at menarche and menopause among Korean women and previous studies [27, 28], we categorized participants as follows: age at menarche (≤ 12, 13–14 [reference], 15, 16, and ≥ 17 years), age at menopause (< 40, 40–45, 46–50, 51–54 [reference], and ≥ 55 years), and reproductive span (< 30, 30–33, 34–36, 37–40 [reference], and ≥ 41 years). The restricted cubic spline curve supported our categorization for each reproductive factor in association with CVD risk (Additional file 2: Fig. S1). Information regarding parity (0, 1, or ≥ 2 children), total lifetime breast feeding history (never, < 6, 6–12, or ≥ 12 total months), hormone replacement therapy (HRT) history (never, < 2, 2–5, or ≥ 5 years), and use of oral contraceptives (OC) (never, < 1, or ≥ 1 year) was also collected.

### Study outcomes and follow-up

Endpoints of the study were incident MI and ischemic stroke. Newly diagnosed MI and ischemic stroke cases were identified on the basis of ICD-10 code for MI (ICD-10 codes I21 or I22 during hospitalization or recording of these codes at least twice as an outpatient setting) and ischemic stroke (ICD-10 codes I63 or I64 during hospitalization with claims for brain magnetic resonance imaging or brain computerized tomography) [29]. We defined CVD as the composite of MI and ischemic stroke. The cohort was followed from the health examination date to the date of incident MI, ischemic stroke, death, or until the end of the study period (31 December 2018), whichever came first.

### Covariates: traditional cardiovascular risk factors

Subjects were classified as never, ex-, or current smokers. Based on daily alcohol consumption, alcohol consumption was classified as none (0 g/day), mild to moderate (< 30 g/day), or heavy (≥ 30 g/day) [30, 31]. Regular exercise was defined as performing > 30 min of moderate physical activity at least five times per week or > 20 min of strenuous physical activity at least three times per week. Participants were categorized into five groups based on BMI (kg/m^2^) according to the Asia–Pacific criteria of the World Health Organization as follows: underweight (< 18.5), normal (18.5–23), overweight (23–25), obese (25–30), and severely obese (≥ 30). Baseline comorbidities (hypertension, diabetes mellitus, and dyslipidemia) were identified based on the combination of diagnosis codes (ICD-10) with relevant prescribed medications for each disease and clinical information. Hypertension was defined as a claim with ICD codes I10-I13 or I15 and antihypertensive medication or systolic blood pressure ≥ 140 mmHg or diastolic blood pressure ≥ 90 mmHg. Diabetes mellitus was defined as a claim with E11-E14 codes linked to a history of hypoglycemic medication prescriptions or fasting glucose level ≥ 126 mg/dL. Dyslipidemia was defined as claims with code E78 and lipid-lowering medications or total cholesterol level ≥ 240 mg/dL [29]. Income level was based on the monthly insurance premium because insurance contributions in Korea are based on income level and not health risk.

### Statistical analyses

Baseline values for all variables were measured in 2009. Continuous variables are presented as means ± standard deviations and categorical variables are presented as numbers and percentages. Hazard ratios (HRs) and 95% confidence interval (CI) values for the associations between reproductive factors and MI, ischemic stroke, and composite CVD were analyzed using Cox proportional hazards models. Multivariable-adjusted proportional hazards models were applied. Model 1 included age and traditional cardiovascular risk factors (income, smoking, alcohol consumption, regular exercise, body mass index, systolic blood pressure, total cholesterol, fasting glucose, hypertension, diabetes mellitus, and dyslipidemia). Model 2 additionally included reproductive factors (age at menarche, age at menopause, parity, duration of breast feeding, duration of HRT, and duration of OC use). Model 3 included reproductive span instead of age at menarche and age at menopause. Separate analyses were undertaken for MI and ischemic stroke. To determine which covariates to include for each reproductive factor (age at menarche, age at menopause, and reproductive factor), we drew a directed acyclic graph (DAG) including all covariates (Additional file 3: Fig. S2, Additional file 4: Fig. S3, Additional file 5: Fig. S4). According to the DAG, we assumed that none of the covariates has a causal relationship with age at menarche and CVD. We assumed that age, BMI, income, smoking status, alcohol consumption, regular exercise, age at menarche, parity, duration of breast feeding, and duration of oral contraceptive were common causes of age at menopause and CVD (confounders) and were included in the model. Regarding the association between reproductive span and CVD, we additionally included age at menopause as a confounder. Other variables (hypertension, diabetes mellitus, dyslipidemia, and duration of HRT) were mediators for the association between reproductive span and CVD, and not included in the model. We further examined the associations between various combinations of the three reproductive factors and the incidence for MI and ischemic stroke. We considered age at menarche 13–14 years, age at menopause of 51–54 years, and reproductive span 37–40 years as reference groups. We also conducted a subgroup analysis by comparing participants 60 years or older with those younger than 60 years to take reproductive factors by year of birth into consideration, i.e., the birth-cohort effect [12, 32].

Statistical analyses were performed using SAS version 9.4 (SAS Institute Inc., Cary, NC, USA), and a *P* value < 0.05 was considered statistically significant.

## Results

### Baseline characteristics of the study population

The characteristics of the study participants are presented in Table 1. Participants with incident CVD events were likely to be older (66.9 vs 60.8 years) and to have later menarche (16.8 vs 16.4 years), earlier menopause (49.6 vs 50.1 years), and shorter reproductive span (32.8 vs 33.6 years). There was also a higher proportion of never HRT or ever OC users among those participants with incident CVD than those without incident CVD. Participants with incident CVD had more comorbidities including hypertension, diabetes mellitus, and dyslipidemia than those participants who did not develop CVD.

**Table 1:** Baseline characteristics of the study participants

	Incident cardiovascular disease (myocardial infarction or ischemic stroke)		*P* value	AMD or PR (95% CI)
	No (*n* = 1,163,480)	Yes (*n* = 61,067)		
Age (years)	60.8 ± 8.0	66.9 ± 8.3	< 0.0001	6.16 (6.10–6.23)
Income (quartile)				
Q1 (lowest)	269,311 (23.2)	13,247 (21.7)	< 0.0001	1.00
Q2	219,661 (18.9)	10,941 (17.9)		1.01 (0.99–1.04)
Q3	289,069 (24.9)	14,843 (24.3)		1.04 (1.02–1.07)
Q4 (highest)	385,439 (33.1)	22,036 (36.1)		1.15 (1.13–1.18)
Smoking status				
Never	1,121,142 (96.4)	57,779 (94.6)	< 0.0001	1.00
Ex-smoker	11,914 (1.02)	670 (1.1)		1.09 (1.01–1.17)
Current smoker	30,424 (2.61)	2618 (4.3)		1.62 (1.56–1.68)
Alcohol consumption				
None	1,013,068 (87.1)	54,967 (90.0)	< 0.0001	1.00
Mild	144,190 (12.4)	5827 (9.5)		0.75 (0.74–0.78)
Heavy	6222 (0.50)	273 (0.5)		0.82 (0.73–0.92)
Regular exercise	219,319 (18.9)	8764 (14.4)	< 0.0001	0.73 (0.72–0.75)
Body mass index (kg/m^2^)	24.1 ± 3.1	24.4 ± 3.3	< 0.0001	0.31 (0.28–0.33)
< 18.5	24,611 (2.1)	1582 (2.6)		1.37 (1.31–1.44)
18.5–23	406,916 (35.0)	18,704 (30.6)		1.00
23–25	309,110 (26.6)	15,474 (25.3)		1.08 (1.06–1.11)
25–30	375,149 (32.2)	22,075 (36.2)		1.26 (1.24–1.29)
≥ 30	47,694 (4.1)	3232 (5.29)		1.44 (1.39–1.50)
Systolic BP (mmHg)	125.2 ± 16.1	129.9 ± 17.0	< 0.0001	4.71 (4.58–4.85)
Diastolic BP (mmHg)	76.8 ± 10.1	78.7 ± 10.4	< 0.0001	1.94 (1.86–2.02)
Fasting glucose (mg/dL)	99.0 ± 23.0	105.3 ± 33.8	< 0.0001	6.30 (6.11–6.49)
Total cholesterol (mg/dL)	209.01 ± 43.8	210.1 ± 45.1	< 0.0001	0.96 (0.60–1.32)
Comorbidities				
Hypertension	491,733 (42.3)	37,169 (60.9)	< 0.0001	2.05 (2.01–2.08)
Diabetes mellitus	134,195 (11.5)	13,492 (22.1)	< 0.0001	2.07 (2.03–2.11)
Dyslipidemia	376,475 (32.4)	21,960 (36.0)	< 0.0001	1.16 (1.15–1.18)
Age at menarche (years)	16.4 ± 1.8	16.8 ± 1.8	< 0.0001	0.36 (0.34–0.37)
≤ 12	11,793 (1.0)	373 (0.6)	< 0.0001	0.89 (0.80–0.98)
13–14	146,603 (12.6)	5251 (8.6)		1.00
15	209,389 (18.0)	8718 (14.3)		1.16 (1.12–1.20)
16	244,831 (21.0)	12,700 (20.8)		1.43 (1.38–1.47)
≥ 17	550,864 (47.4)	34,025 (55.7)		1.68 (1.64–1.73)
Age at menopause (years)	50.1 ± 4.0	49.6 ± 4.5	< 0.0001	0.48 (0.45–0.51)
< 40	19,301 (1.7)	1637 (2.7)	< 0.0001	1.88 (1.79–1.98)
40–45	115,815 (10.0)	8154 (13.4)		1.58 (1.54–1.62)
46–50	527,997 (45.4)	28,694 (47.0)		1.24 (1.22–1.26)
51–54	434,332 (37.3)	18,815 (30.8)		1.00
≥ 55	66,035 (5.7)	3767 (6.2)		1.30 (1.26–1.34)
Reproductive span (years)	33.6 ± 4.4	32.8 ± 4.9	< 0.0001	0.84 (0.80–0.88)
< 30	156,340 (13.4)	11,567 (18.9)	< 0.0001	1.79 (1.74–1.84)
30–33	351,323 (30.2)	20,109 (32.9)		1.41 (1.37–1.44)
34–36	471,279 (40.5)	21,244 (34.8)		1.15 (1.13–1.18)
37–40	161,421 (13.9)	6737 (11.0)		1.00
≥ 41	23,117 (2.0)	1410 (2.3)		1.37 (1.30–1.43)
Parity				
Nulliparity	28,295 (2.4)	990 (1.6)	< 0.0001	1.00
1	1,062,402 (91.3)	57,740 (94.6)		0.92 (0.86–0.99)
≥ 2	72,783 (6.3)	2337 (3.8)		1.52 (1.43–1.62)
Duration of breast feeding (months)				
Never	78,590 (6.8)	2410 (4.0)	< 0.0001	1.00
< 6	77,652 (6.7)	2195 (3.6)		0.92 (0.87–0.98)
6–12	203,318 (17.5)	7776 (12.7)		1.24 (1.18–1.30)
≥ 12	803,920 (69.1)	48,686 (79.7)		1.92 (1.84–2.00)
Duration of HRT (years)				
Never	971,399 (83.5)	54,552 (89.3)	< 0.0001	1.00
< 2	112,034 (9.6)	3709 (6.1)		0.60 (0.58–0.62)
2–5	45,869 (3.9)	1537 (2.5)		0.61 (0.58–0.64)
≥ 5	34,178 (2.9)	1269 (2.1)		0.67 (0.64–0.71)
Duration of OC use (years)				
Never	978,738 (84.1)	52,483 (85.9)	< 0.0001	1.00
< 1	111,850 (9.6)	4992 (8.2)		0.84 (0.82–0.86)
≥ 1	72,892 (6.3)	3592 (5.9)		0.92 (0.89–0.95)

Data are expressed as means ± SD or *n* (%)

*AMD* absolute mean difference, *PR* prevalence ratio, *CI* confidence interval, *BP* blood pressure, *HRT* hormone replacement therapy, *OC* oral contraceptive

### Associations between age at menarche, age at menopause, and reproductive span with CVD events

During a median follow-up of 8.4 years, 61,067 CVD events (25,181 MI and 38,996 ischemic stroke cases) were identified. Deaths during follow-up until the end of the study were 50,990 cases (4.2%) and the rate of dropping out from NHIS due to immigration is low (less than 2300 cases per year). Late menarche was associated with an increased risk of MI compared to 13–14 years at menarche: 16 years (aHR 1.06, 95% CI 1.01–1.11) and ≥ 17 years (aHR 1.06, 95% CI 1.02–1.11) (Table 2, Additional file 6: Fig. S5). Compared to women who experienced menopause at 51–54 years, early menopause was associated with an increased risk of MI: 46–50 years (aHR 1.12, 95% CI 1.09–1.16), 45–45 years (aHR 1.24, 95% CI 1.19–1.29), and < 40 years (aHR 1.40, 95% CI 1.30–1.52). When compared to a reproductive span of 37–40 years, a shorter reproductive span was associated with an increased risk of MI: 34–36 years (aHR 1.12, 95% CI 1.08–1.16), 30–33 years (aHR 1.19, 95% CI 1.15–1.16), and < 30 years (aHR 1.32, 95% CI 1.26–1.38).

**Table 2:** Hazard ratios and 95% confidence intervals of myocardial infarction, ischemic stroke, and cardiovascular disease according to reproductive factors (age at menarche, age at menopause, and reproductive span)

Reproductive factors	Subjects (*N*)	**Myocardial infarction**				**Ischemic stroke**				**Cardiovascular disease**			
		Events (*n*)	IR	Model 1	Model 2	Events (*n*)	IR	Model 1	Model 2	Events (*n*)	IR	Model 1	Model 2
**Age at menarche (years)**													
≤ 12	12,166	137	1.4	0.86 (0.72–1.02)	0.86 (0.72–1.02)	252	2.5	**1.15 (1.01–1.31)**	**1.16 (1.02–1.31)**	373	3.7	1.03 (0.93–1.14)	1.03 (0.93–1.15)
13–14	151,854	2267	1.8	1 (Ref.)	1 (Ref.)	3238	2.6	1 (Ref.)	1 (Ref.)	5251	4.2	1 (Ref.)	1 (Ref.)
15	218,107	3712	2.1	1.02 (0.97–1.07)	1.01 (0.96–1.07)	5418	3.0	1.01 (0.96–1.05)	1.00 (0.96–1.04)	8718	4.9	1.01 (0.98–1.05)	1.01 (0.97–1.04)
16	257,531	5268	2.5	**1.07 (1.01–1.12)**	**1.06 (1.01–1.11)**	8074	3.8	**1.08 (1.03–1.12)**	**1.07 (1.03–1.11)**	12,700	6.1	**1.08 (1.04–1.11)**	**1.07 (1.03–1.10)**
≥ 17	584,889	13,797	2.9	**1.08 (1.03–1.13)**	**1.06 (1.02–1.11)**	22,014	4.6	**1.11 (1.07–1.15)**	**1.09 (1.05–1.13)**	34,025	7.2	**1.10 (1.07–1.13)**	**1.08 (1.05–1.12)**
*P* for trend				< 0.0001	0.0002			< 0.0001	< 0.0001			< 0.0001	< 0.0001
**Age at menopause (years)**													
< 40	20,938	686	4.0	**1.41 (1.30–1.52)**	**1.40 (1.30–1.52)**	1048	6.2	**1.32 (1.24–1.40)**	**1.31 (1.23–1.39)**	1637	9.8	**1.36 (1.29–1.43)**	**1.35 (1.28–1.42)**
40–45	123,969	3372	3.3	**1.24 (1.19–1.30)**	**1.24 (1.19–1.29)**	5246	5.2	**1.18 (1.15–1.22)**	**1.18 (1.14–1.22)**	8154	8.2	**1.21 (1.18–1.24)**	**1.21 (1.17–1.24)**
46–50	556,691	11,771	2.6	**1.12 (1.09–1.16)**	**1.12 (1.09–1.16)**	18,400	4.1	**1.12 (1.09–1.14)**	**1.12 (1.09–1.14)**	28,694	6.4	**1.12 (1.10–1.14)**	**1.12 (1.10–1.14)**
51–54	453,147	7833	2.1	1 (Ref.)	1 (Ref.)	11,860	3.2	1(Ref.)	1 (Ref.)	18,815	5.1	1 (Ref.)	1 (Ref.)
≥ 55	69,802	1519	2.6	0.99 (0.93–1.04)	0.98 (0.93–1.04)	2442	4.3	1.01(0.97–1.06)	1.01 (0.97–1.05)	3767	6.7	1.00 (0.96–1.03)	1.00 (0.96–1.03)
*P* for trend				< 0.0001	< 0.0001			< 0.0001	< 0.0001			< 0.0001	< 0.0001
**Reproductive span (years)**^**a**^													
< 30	167,907	4735	3.5	**1.33 (1.27–1.39)**	**1.32 (1.26–1.38)**	7495	5.5	**1.27 (1.23–1.32)**	**1.26 (1.22–1.31)**	11,567	8.6	**1.30 (1.26–1.33)**	**1.29 (1.25–1.32)**
30–33	371,432	8250	2.7	**1.20 (1.16–1.25)**	**1.19 (1.15–1.24)**	12,909	4.3	**1.17 (1.14–1.21)**	**1.17 (1.13–1.20)**	20,109	6.7	**1.19 (1.16–1.22)**	**1.18 (1.15–1.21)**
34–36	401,857	7444	2.2	**1.12 (1.08–1.17)**	**1.12 (1.08–1.16)**	11,298	3.4	**1.10 (1.07–1.14)**	**1.10 (1.06–1.13)**	17,877	5.5	**1.11 (1.08–1.14)**	**1.11 (1.08–1.13)**
37–40	240,950	3861	1.9	1 (Ref.)	1 (Ref.)	5842	2.9	1 (Ref.)	1 (Ref.)	9284	4.7	1 (Ref.)	1 (Ref.)
≥ 41	42,401	891	2.6	1.03 (0.96–1.11)	1.03 (0.96–1.11)	1452	4.2	**1.06 (1.00–1.12)**	**1.06 (1.00–1.13)**	2230	6.5	1.05 (1.00–1.10)	**1.05 (1.00–1.10)**
*P* for trend				< 0.0001	< 0.0001			< 0.0001	< 0.0001			< 0.0001	< 0.0001

*IR* incidence rate per 1000 person-years

Model 1: The full model included age and traditional cardiovascular risk factors (income, smoking, alcohol consumption, regular exercise, body mass index, systolic blood pressure, total cholesterol, fasting glucose, hypertension, diabetes mellitus, and dyslipidemia)

Model 2: The full model included age, traditional cardiovascular risk factors (income, smoking, alcohol consumption, regular exercise, body mass index, systolic blood pressure, total cholesterol, fasting glucose, hypertension, diabetes mellitus, and dyslipidemia), and reproductive factors (age at menarche, age at menopause, parity, duration of breast feeding, duration of hormone replacement therapy, and duration of oral contraceptive use)

^a^The full model included reproductive span instead of age at menarche and menopause in model 2

Values in bold indicate statistical significance

In contrast, different associations were observed between age at menarche, reproductive span, and ischemic stroke (Table 2). Both early menarche (≤ 12 years, aHR 1.16, 95% CI 1.02–1.31) and late menarche (16 years, aHR 1.07, 95% CI 1.03–1.11; ≥ 17 years, aHR 1.09, 95% CI 1.05–1.13) were associated with an increased risk of ischemic stroke, compared to 13–14 years at menarche, yielding a U-shaped association between age at menarche and ischemic stroke. In addition, compared to a reproductive span of 37–40 years, both shorter (≤ 36 years) and longer (≥ 41 years) reproductive spans were associated with an increased risk of ischemic stroke: < 30 years (aHR 1.26, 95% CI 1.22–1.31), 30–33 years (aHR 1.17, 95% CI 1.13–1.20), 34–36 years (aHR 1.10, 95% 1.03–1.13), and ≥ 41 years (aHR 1.06, 95% CI 1.00–1.13).

In the sensitivity analysis, (1) the results without lag time showed consistent associations with the main analysis (Additional file 1: Table S3) and (2) the results from the model, which consists of confounders from DAG, showed a consistent association with the main findings while attenuated (Additional file 1: Table S4).

### Associations between combinations of the three reproductive factors and CVD risk

**Fig. 2:**
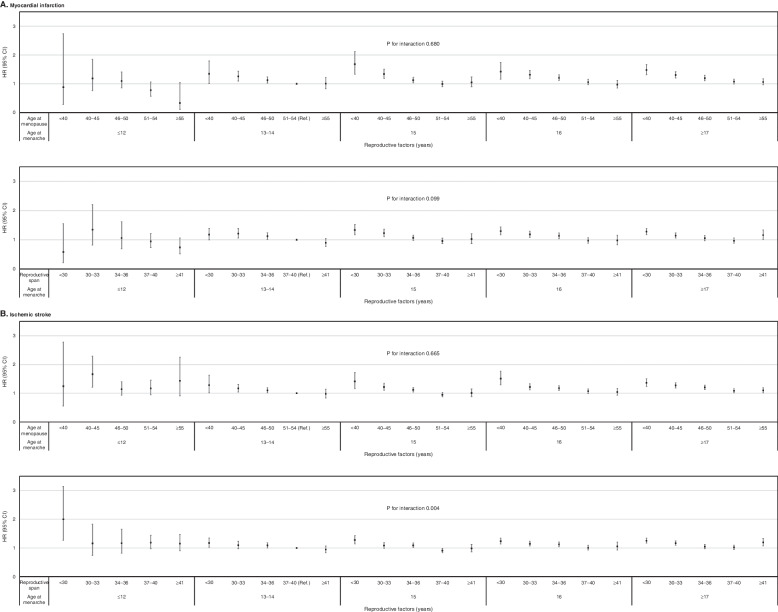
Associations between age at menarche in combination with age at menopause and reproductive span and the risk of cardiovascular events. HR, hazard ratio adjusted for cardiovascular risk factors (income, smoking, alcohol consumption, regular exercise, body mass index, systolic blood pressure, total cholesterol, fasting glucose, hypertension, diabetes mellitus, and dyslipidemia) and reproductive factors (parity, duration of breast feeding, duration of hormone replacement therapy, and duration of oral contraceptive use); CI, confidence interval, **a**) the association of combined reproductive factors with myocardial infarction. **b**) the association of combined reproductive factors with ischemic stroke

The combination of late menarche and early menopause (< 40 years) was associated with an increased risk of MI: 15 years (aHR 1.69, 95% CI 1.34–2.13), 16 years (aHR 1.44, 95% CI 1.17–1.76), and ≥ 17 years (aHR 1.50, 95% CI 1.33–1.70) (*P* for interaction = 0.68). Similarly, the combination of a short reproductive span (< 30 years) and late menarche increased the risk of MI: 15 years (aHR 1.34, 95% CI 1.17–2.13), 16 years (aHR 1.44, 95% CI 1.17–1.76), and ≥ 17 years (aHR 1.50, 95% CI 1.33–1.70) (*P* for interaction = 0.09). There was a significant interaction between reproductive span and age at menarche for ischemic stroke (*P* for interaction = 0.004). Increased risk of ischemic stroke related to short reproductive span (< 30 years) was strengthened by early age at menarche (≤ 12 years) (aHR 2.00, 95% CI 1.27–3.14). A U-shaped association between age at menarche and risk of ischemic stroke was observed compared to the reference group. The combination of either early or late menarche with a short reproductive span and early menopause was associated with an increased risk of ischemic stroke (Fig. 2 and Additional file 1: Table S2).

### Age-stratified analyses

Age-stratified analyses (< 60 years and ≥ 60 years) yielded results consistent with those obtained for the entire study population (Additional file 1: Table S3). The increased risk of MI associated with late menopause and early menopause and short reproductive span was more prominent in the younger age group (< 60 years) than the older age group (≥ 60 years).

## Discussion

In this large nationwide study, we investigated the association between reproductive factors and risk of MI and ischemic stroke. We found that late menarche, early menopause, and short reproductive span duration were associated with an increased risk of MI. Meanwhile, a U-shaped association between age at menarche and risk of ischemic stroke was noted, with a 16% higher risk in early menarche (≤ 12 years) and a 7–9% higher risk in late menarche (≥ 16 years). Similarly, both shorter and longer duration reproductive spans were associated with an increased risk of ischemic stroke.

Various patterns of association between age at menarche and CVD incidence have been reported. We found that early menarche was associated with an increased risk of ischemic stroke, but not MI, unlike several pre-existing studies that found a higher risk of both CHD and stroke for those women who had experienced early menarche [6–8, 13]. One possible reason for our findings is that the etiology of MI is largely uniform, namely atherosclerotic plaque rupture with superimposed in situ arterial thrombosis, whereas the etiology of ischemic stroke is heterogenous involving atherothrombotic, cardioembolic, small-vessel disease, and cryptogenic causes [33]. Although estrogen protects against atherosclerosis, it can increase coagulation by activating intrinsic pathway factors thereby increasing the risk of ischemic stroke, but not MI [34].

We found that late menarche was associated with an increased risk of both MI and ischemic stroke. A similar finding was reported in a previous study of Korean women: menarche ≥ 17 years was associated with an aHR of 1.62 (95% CI 1.11–2.36) for MI and an aHR of 1.22 (95% CI 1.09–1.36) for stroke, whereas most other studies have reported no association [6–8, 13]. This difference may partly be explained by differences in disease epidemiology among countries. In Korea, the incidence of ischemic stroke is higher than that of MI (63.3 vs 40.0 per 100,000 person-years in 2013) [35, 36]. In addition, late menarche could be related to low BMI and poor nutrition during puberty. Underweight adolescents were expected to have late menarche (HR 0.27, 95% CI 0.12–0.60), which in turn leads to a higher risk of CVD in adults [37]. In particular, the incidence of underweight is more prevalent in Asian than Western populations [38]. However, late menarche may be linked to higher prepubertal cortisol secretion levels through inhibition of pulsatile luteinizing hormone due to suppression of hypothalamic-pituitary responsiveness to GnRH [39, 40]; high cortisol levels are known to increase the incidence and progression of atherogenesis [41].

In agreement with previous studies [6–11], early age at menopause was associated with an increased risk of both MI and ischemic stroke. A pooled analysis of observational studies reported an increased risk of non-fatal CVD in women who had undergone premature menopause (< 40 years; HR 1.55, 95% CI 1.37–1.73) and early menopause (age 40 to 44 years; HR 1.30, 95% CI 1.22–1.39) [42]. An early decrease in endogenous estrogens adversely affects cardiovascular risk factors such as lipid and blood pressure, negating its protective effects as an endothelial vasodilator.

 The association between a shorter reproductive span and a higher risk of MI or ischemic stroke is consistent with the association between age at menopause and MI and ischemic stroke, because the reproductive span is largely determined by age at menopause. Furthermore, a short reproductive span is associated with an increased risk of atrial fibrillation, which in turn can increase the risk of ischemic stroke [43]. We also found that a longer reproductive span was associated with an increased risk of ischemic stroke, but not of MI. The reason for this is unclear, but it may be partly associated with the increased risk of stroke in very early menarche (< 12 years).

The combination of late menarche and early menopause/reproductive span increased the risk of MI in an additive manner, with the lowest risk found in those participants with early menarche and late menopause/long reproductive span. Interestingly, early menarche increased the risk of ischemic stroke regardless of age at menopause and reproductive span in contrast to the associations between these factors and MI [19]. Women who had undergone early menarche and had a short reproductive span had the highest risk of ischemic stroke, perhaps due to the combined effect of improper timing of estrogen production and insufficient estrogen exposure.

The association between reproductive factors and CVD risk was stronger in women less than 60 years compared to women aged ≥ 60 years. This interesting finding may be attributed to the different CVD risks of each reference group. Old age itself is a potent risk factor for CVD risk, and the old age group is more likely to have other traditional risk factors (e.g., hypertension), which results in the smaller effect size of reproductive factors in old-aged women.

Clinical guidelines for primary prevention of CVD in women acknowledge premature menopause (< 40 years) as a risk-enhancing factor in addition to traditional CVD risk factors and suggest that statin therapy be considered in those women who undergo premature menopause [42]. However, the relationships between age at menarche and reproductive span and CVD are still inconsistent and further studies are needed to investigate the relationship between these reproductive factors and the risk of MI and ischemic stroke.

Our study has several strengths including the large study population, adjustment for various reproductive factors besides traditional CVD risk factors, and evaluation of the association between various combinations of the reproductive factors of age at menarche, age at menopause, and reproductive span and MI and ischemic stroke risk. Furthermore, even though age at menarche and age at menopause are strongly interrelated [44], only a few studies have adjusted for both age at menarche and age at menopause, unlike our study [8–11]. However, several limitations need to be considered when interpreting our findings. First, there may have been a recall bias associated with self-reported age at menarche and age at menopause. We consider this unlikely though as a previous study reported a high correlation (*r* = 0.79) and mean absolute error of 0.62 years between the recalled and original age at menarche [45]. Second, we were not able to include the information on body weight or nutrition status at adolescents even though we suggested underweight as a key factor. Particularly, a dietary pattern is an important confounding factor for the association between reproductive factor and CVD, which could not be considered on this study [46–48]. Further studies including such confounding factor are needed. Third, we included only Korean women; thus, the generalizability of our study findings is limited. Racial/ethnic differences may exist in the associations between reproductive factors and CVD risk because reproductive trajectories vary across different races/ethnicities [49]. Fourth, we did not include the women with established CVD because of concerns about accuracy in identifying the recurrent CVD events. Further studies are needed with a sophisticated operational definition of recurring cases from the viewpoints of secondary prevention.

## Conclusions

In conclusion, we demonstrated different patterns of association between age at menarche and MI and ischemic stroke. While late menarche was linearly associated with an increased risk of MI, both early and late menarche were associated with an increased risk of ischemic stroke. Early menopause and short reproductive span were linearly associated with an increased risk of both MI and ischemic stroke, except for a slight increase in the risk of ischemic stroke in women with a reproductive span longer than 40 years. Clinicians need to consider female reproductive factors in addition to traditional CVD risk factors when assessing the overall risk of CVD in postmenopausal women.

## Supplementary Information

**Additional file 1: Supplementary Tables. Table S1.** A summary of previous studies (selected) on the relationship between reproductive factors and cardiovascular disease incidence. **Table S2.** Information on missing data. **Table S3. **Hazard ratios and 95% confidence intervals of myocardial infarction, ischemic stroke, and cardiovascular disease according to reproductive factors (age at menarche, age at menopause, and reproductive span) without lag periods. **Table S4. **Hazard ratios and 95% confidence intervals of myocardial infarction, ischemic stroke, and cardiovascular disease according to reproductive factors (age at menarche, age at menopause, and reproductive span) adjustment for confounders based on directed acyclic graph. **Table S5. **Age at menarche in combination with age at menopause and reproductive span and the risk of cardiovascular events.** Table S6. **Hazard ratios and 95% confidence intervals of cardiovascular disease by reproductive factors according to age group.

**Additional file 2: Fig. S1. **The restricted cubic spline curve for the association of reproductive factors with cardiovascular diseases.

**Additional file 3: Fig. S2. **Directed acyclic graph illustrating the assumptions about the causal relationship between age at menarche and cardiovascular disease. Red circles indicate confounders, blue circles represent mediators/colliders. The grey circle refers to an unmeasured variable. BMI, body mass index; HRT, hormone replacement therapy; CVD, cardiovascular disease

**Additional file 4: Fig. S3. **Directed acyclic graph illustrating the assumptions about the causal relationship between age at menopause and cardiovascular disease. Red circles indicate confounders, blue circles represent mediators/colliders. The grey circle refers to an unmeasured variable. BMI, body mass index; HRT, hormone replacement therapy; CVD, cardiovascular disease.

**Additional file 5: Fig. S4. **Directed acyclic graph illustrating the assumptions about the causal relationship between reproductive span and cardiovascular disease. Red circles indicate confounders, blue circles represent mediators/colliders. The grey circle refers to an unmeasured variable. BMI, body mass index; HRT, hormone replacement therapy; CVD, cardiovascular disease.

**Additional file 6: Fig. S5. **Hazard ratios (HRs) and 95% confidence intervals (CIs) for cardiovascular events according to menstrual history. ^a^Model 2: The full model included age, cardiovascular risk factors (income, smoking, alcohol consumption, regular exercise, body mass index, systolic blood pressure, total cholesterol, fasting glucose, hypertension, diabetes mellitus, and dyslipidemia) and reproductive factors (age at menarche, age at menopause, parity, duration of breast feeding, duration of hormone replacement therapy, and duration of oral contraceptive use). ^b^Model 3: The full model included age, cardiovascular risk factors (income, smoking, alcohol consumption, regular exercise, body mass index, systolic blood pressure, total cholesterol, fasting glucose, hypertension, diabetes mellitus, and dyslipidemia) and reproductive factors (reproductive span, parity, duration of breast feeding, duration of hormone replacement therapy, and duration of oral contraceptive use)

## Data Availability

The data that support the findings of this study are available from the Korean National Health Insurance Service (KNHIS) and were used under license for the current study (http://nhiss.nhis.or.kr). Restrictions apply to their availability (data are not publicly available). Data are available with permission of the KNHIS from the authors upon reasonable request.

## Abbreviations

aHR: Adjusted hazard ratio
BMI: Body mass index
CHD: Coronary heart disease
CI: Confidence interval
CVD: Cardiovascular disease
DAG: Directed acyclic graph
HRs: Hazard ratios
HRT: Hormone replacement therapy
ICD-10: International Classification of Disease 10th revision
IS: Ischemic stroke
MI: Myocardial infarction
NCSP: National Cancer Screening Program
NHIS: National Health Insurance Services
OC: Oral contraceptives
