# Supplementary material for: Associations of reproductive factors with incidence of myocardial infarction and ischemic stroke in postmenopausal women: a cohort study
Source: BMC Med. 2023 Feb 20;21:64. doi: 10.1186/s12916-023-02757-2 (PMC9942298; doi:10.1186/s12916-023-02757-2)
Supplement: Supplementary file 1 — Additional file 1: Supplementary Tables. Table S1. A summary of previous studies (selected) on the relationship between reproductive factors and cardiovascular disease incidence. Table S2. Information on missing data. Table S3. Hazard ratios and 95% confidence intervals of myocardial infarction, ischemic stroke, and cardiovascular disease according to reproductive factors (age at menarche, age at menopause, and reproductive span) without lag periods. Table S4. Hazard ratios and 95% confidence intervals of myocardial infarction, ischemic stroke, and cardiovascular disease according to reproductive factors (age at menarche, age at menopause, and reproductive span) adjustment for confounders based on directed acyclic graph. Table S5. Age at menarche in combination with age at menopause and reproductive span and the risk of cardiovascular events. Table S6. Hazard ratios and 95% confidence intervals of cardiovascular disease by reproductive factors according to age group. [file 12916_2023_2757_MOESM1_ESM.docx]

**Table S1** A summary of previous studies (selected) on the relationship between reproductive factors and cardiovascular disease incidence.

| **Cohort Study** | | | | | | | | | | | | | |
| --- | --- | --- | --- | --- | --- | --- | --- | --- | --- | --- | --- | --- | --- |
| **Study** | **Participants’ Country** | **No. of**  **participants** | **Range of age**  **(years)** | **Follow-up**  **(years)** | **Adjustment** | **Outcomes** | **No. of**  **outcomes** | **Age at menarche (years)**  **[HR (95%CI)]** | | **Age at menopause (years)**  **[HR (95%CI)]** | | **Reproductive span (years)**  **[HR (95%CI)]** | |
| Peters et al.  (2018) | Pre- and post- menopausal women,  UK | 267,440 | 40-69 | 7 | age, socioeconomic status, smoking, SBP, BMI, history of DM | coronary  heart disease (ICD-10 codes I21, I22, I23, I24.1 or I25.2) | 1,577 | < 12  12  13  14  ≥ 15 | 1.08 (0.97-1.20)  1.04 (0.93-1.17)  1.00 (ref)  1.03 (0.92-1.15)  1.04 (0.93; 1.18) | < 47  47-49  50  51-52  53-54  ≥ 55 | **1.33 (1.15-1.53)**  **1.26 (1.08-1.47)**  1.00 (ref)  0.85 (0.72-1.00)  1.12 (0.94-1.33)  0.99 (0.85-1.17) | N/A | |
|  |  |  |  |  |  | stroke  (ICD-10 codes I60-I61, I63-I64) | 1,453 | < 12  12  13  14  ≥ 15 | **1.17 (1.05-1.31)**  1.00 (0.89-1.13)  1.00 (ref)  1.00 (0.88-1.12)  1.02 (0.90-1.16) | < 47  47-49  50  51-52  53-54  ≥ 55 | **1.55 (1.34-1.78)**  1.12 (0.94-1.34)  1.00 (ref)  1.14 (0.98-1.32)  1.06 (0.88-1.28)  1.13 (0.96-1.32) | N/A | |
| Ley et al.  (2017) | Pre- and post- menopausal women,  US | 73,814 | 30-55 | 19 | age, ethnicity, smoking, alcohol, exercise, Alternate Healthy Eating Index, BMI, DM, HTN, DL, aspirin use, family history of MI/stroke, menopause type ^b^, parity, HRT, OC | coronary  heart disease (ICD-10 codes N/A) | 3,254 | ≤ 10  11  12  13  14  15  ≥ 16 | 1.16 (1.00-1.34)  1.00 (0.90-1.12)  1.00 (0.91-1.09)  1.00 (ref)  1.03 (0.91-1.15)  0.88 (0.73-1.05)  1.05 (0.87-1.26) | < 40  40-44  45-49  50-54  ≥ 55 | **1.37 (1.14–1.63)**  **1.22 (1.06–1.40)**  **1.14 (1.05–1.24)**  1.00 (ref)  0.96 (0.83–1.12) | < 30  30-33  34-37  38-41  ≥ 42 | **1.39 (1.17-1.65)**  **1.24 (1.06-1.45)**  1.13 (0.99-1.29)  1.05 (0.93-1.20)  1.00 (ref) |
|  |  |  |  |  |  | stroke  (ICD-10 codes N/A) | 2,684 | ≤ 10  11  12  13  14  15  ≥ 16 | **1.25 (1.07-1.46)**  0.96 (0.86-1.07)  0.95 (0.86-1.05)  1.00 (ref)  1.06 (0.94-1.18)  0.87 (0.72-1.04)  1.02 (0.84-1.22) | < 40  40-44  45-49  50-54  ≥ 55 | **1.25 (1.04–1.51)**  **1.23 (1.07–1.42)**  1.00 (0.91–1.09)  1.00 (ref)  1.09 (0.94–1.26) | < 30  30-33  34-37  38-41  ≥ 42 | **1.27 (1.06-1.52)**  1.09 (0.93-1.28)  1.01 (0.89-1.16)  1.02 (0.89-1.16)  1.00 (ref) |
| Yang et al.  (2017) | * Post- menopausal women,  China | 155,946 | ≤ 79 | 7 | age, education, region, income, smoking, alcohol, exercise, BP, BMI, DM, parity, age at first birth, breastfeeding, OC | coronary  heart disease  (ICD-10 codes I20-I25) | N/A | ≤ 12  13  14  15  16  17  ≥ 18 | 0.99 (0.90-1.09)  1.00 (0.94-1.07)  **1.09 (1.03-1.14)**  1.04 (1.00-1.09)  0.98 (0.94-1.02)  1.04 (1.00-1.09)  0.97 (0.93-1.01) | N/A | | N/A | |
|  |  |  |  |  |  | cerebrovascular disease (ICD-10 codes I60-I69) | N/A | ≤ 12  13  14  15  16  17  ≥ 18 | 1.05 (0.96, 1.16)  1.00 (ref)  **1.07 (1.02, 1.12)**  **1.06 (1.02, 1.11)**  **1.05 (1.01, 1.09)**  1.02 (0.98, 1.07)  1.00 (0.96, 1.04) | N/A | | N/A | |
| Yang et al.  (2017) | * Post- menopausal women,  China | 134,010 | Mean  58.9 | 9 | age, education, income, smoking, alcohol, exercise, BP, BMI, DM, age at menarche, parity, number of abortions, age at first birth, breastfeeding, OC | coronary  heart disease  (ICD-10 codes I20-I25) | 13,465 | N/A | | < 43  43-47  48-50  51-52  ≥ 53 | 1.06 (N/A)  1.03 (N/A)  1.00 (ref)  0.98 (N/A)  0.97 (N/A) | < 30  30-32  33-35  ≥ 36 | 1.04 (N/A)  1.00 (ref)  1.01 (N/A)  0.97 (N/A) |
|  |  |  |  |  |  | stroke  (ICD-10 codes I60-I61, I63-I64) | 14,368 | N/A | | < 43  43-47  48-50  51-52  ≥ 53 | 1.06 (N/A)  1.02 (N/A)  1.00 (ref)  0.95 (N/A)  0.99 (N/A) | < 30  30-32  33-35  ≥ 36 | 1.06 (N/A)  1.00 (ref)  0.99 (N/A)  0.99 (N/A) |
| Jung et al.  (2016) | Pre- and post- menopausal women,  Korea | 66,104 | Mean  47.3 | 12.4 | age, socioeconomic status, exercise, SBP, BMI, TC, HDL, DM, age at menarche ^b^ | acute myocardial infarction  (ICD-10 codes N/A) | 249 | ≤ 12  13-14  15-16  ≥ 17 | 1.13 (0.44-2.86)  1.00 (ref)  1.42 (0.99-2.05)  **1.62 (1.11-2.36)** | ≤ 45  46-48  49-51  ≥ 52 | 1.39 (0.87-2.22)  1.37 (0.86-2.19)  1.00 (ref)  0.95 (0.60-1.49) | < 30 30-35  36-39  ≥ 40 | **1.73 (1.08-2.79)**  **1.64 (1.08-2.47)**  1.00 (ref)  1.68 (0.87-3.24) |
|  |  |  |  |  |  | cerebrovascular disease (ICD-10 codes I60-I69) | 2,446 | ≤ 12  13-14  15-16  ≥ 17 | 0.80 (0.61-1.06)  1.00 (ref)  1.03 (0.93-1.15)  **1.22 (1.09-1.36)** | ≤ 45  46-48  49-51  ≥ 52 | 1.05 (0.89-1.24)  1.14 (0.97-1.34)  1.00 (ref)  0.90 (0.77-1.04) | < 30 30-35  36-39  ≥ 40 | 1.18 (1.00-1.38)  **1.15 (1.01-1.32)**  1.00 (ref)  0.90 (0.70-1.17) |
| Murakami et al.  (2016) | Post-menopausal women,  Japan | 1,412 | ≥ 35 | 12.8 | age, smoking, alcohol, height, BMI, DM, HTN, DL, heart disease, menopause type, age at menopause ^a^, age at menarche ^b^, parity, HRT | stroke  (ICD-10 codes N/A) | 143 | ≤ 13  14  15  ≥ 16 | **1.83 (1.04-3.22)**  1.32 (0.74-2.35)  1.00 (ref)  1.14 (0.68-2.35) | ≤ 45  46-49  50  ≥ 51 | 1.68 (0.92-3.06)  **1.76 (1.05-2.94)**  1.00 (ref)  1.20 (0.70-2.04) | N/A | |
| Canoy et al.  (2014) | Post-menopausal women,  UK | 1,217,840 | 50-64 | 11.6 | age, socioeconomic status, smoking, alcohol, exercise, height, BMI, DM, HTN, DL, menopausal status, parity, HRT, OC | coronary  heart disease  (ICD-10 codes I20-I25) | 73,378 | ≤ 10  11  12  13  14  15  16  ≥ 17 | **1.27 (1.22-1.31)**  **1.12 (1.10-1.14)**  **1.02 (1.01-1.04)**  1.00 (ref)  **1.04 (1.02-1.06)**  **1.06 (1.04-1.08)**  **1.10 (1.07-1.14)**  **1.23 (1.16-1.29)** | N/A | | N/A | |
|  |  |  |  |  |  | cerebrovascular disease (ICD-10 codes I60–I69) | 25,426 | ≤ 10  11  12  13  14  15  16  ≥ 17 | **1.16 (1.09-1.23)**  **1.04 (1.01-1.07)**  1.00 (0.97-1.03)  1.00 (ref)  1.00 (0.97-1.02)  1.01 (0.97-1.05)  1.06 (1.00-1.12)  **1.13 (1.03-1.24)** | N/A | | N/A | |
| Lakshman et al.  (2009) | Post-menopausal women,  UK | 15,807 | 40-79 | 10.6 | age, education, occupation, smoking, alcohol, exercise, BMI, WC, parity, HRT, OC | cardiovascular disease  (ICD-10 codes I10–I79) | 3,888 | 8-11  12  13  14  15-18 | 1.00 (ref)  **0.83 (0.74–0.92)**  **0.79 (0.72–0.87)**  **0.83 (0.75–0.91)**  **0.84 (0.76–0.93)** | N/A | | N/A | |
| Hu et al.  (1999) | Post-menopausal women,  US | 35,616 | 30-55 | 9.9 | age, smoking, DM, HTN, DL, parental history of premature MI, age at menarche, parity | coronary  heart disease  (ICD-10 code N/A) | 757 | N/A | | < 40  40-44  45-49  50-54  ≥ 55 | 1.53 (0.94-2.47)  **1.42 (1.08-1.86)**  1.10 (0.93-1.29)  1.00 (ref)  0.95 (0.67-1.34) | N/A | |
|  |  |  |  |  |  | stroke  (ICD-10 code N/A) | 350 | N/A | | < 40  40-44  45-49  50-54  ≥ 55 | 0.74 (0.27-2.00)  0.95 (0.60-1.51)  1.13 (0.89-1.43)  1.00 (ref)  0.98 (0.61-1.58) | N/A | |
| **Systemic review** | | | | | | | | | | | | | |
| **Study** | **Included studies** | **No. of**  **Participants** | | | | **Outcomes** | **No. of**  **outcomes** | **Age at menarche** | | **Age at menopause** | | **Reproductive span** | |
| Okoth et al.  (2020) | Umbrella review  (32 review studies) | 50,125 | | | | coronary  heart disease  (ICD-10 code N/A) | 1,217 | N/A | | Early menopause (< 45)  RR **1.50 (1.28-1.76)** | | N/A | |
|  |  | 49,246 | | | | stroke  (ICD-10 code N/A) | 770 | N/A | | Early menopause (< 45)  RR 1.23 (0.98-1.53) | | N/A | |
|  |  | 121,800 | | | | cardiovascular disease  (ICD-10 code N/A) | 2,264 | Early menarche (<12)  HR **1.15 (1.02-1.28)** | | N/A | | N/A | |
| Mishra et al.  (2021) | Meta analysis  (17 cohort studies) | 14,285 (age at menarche)  4,144 (age at menopause)  585,386 (reproductive lifespan) | | | | stroke  (ICD-10 code N/A) | N/A | Early menarche  RR 1.17 (0.20, 2.14) | | Early menopause  RR 1.13 (0.71, 1.56) | | Short reproductive lifespan  RR **1.31 (1.25-1.36)** | |
| Mishra et al.  (2020) | Pooled analysis  (12 studies) | 307,855  Post- menopausal women | | | age, race, education, smoking, BMI, parity, age at first birth, HRT | coronary  heart disease  (ICD-10 codes I20-I25) | 7,610 | ≤10 11  12  13  14  15 ≥16 | 1.09 (0.96-1.24)  **1.16 (1.07-1.25)**  0.97 (0.90-1.04)  1.00 (ref)  0.96 (0.90-1.03)  1.01 (0.94-1.10)  **1.11 (1.01-1.21)** | < 40 40-44 45-49 50-51 52-53 54-55  ≥ 56 | **1.89 (1.64-2.19)**  **1.57 (1.44-1.72)**  **1.22 (1.14-1.31**)  1.00 (ref)  0.93 (0.86-1.01)  **0.80 (0.75-0.90)**  **0.72 (0.65-0.81)** | < 30  30-32 33-35 36-38 39-41 42-44  ≥ 45 | **1.66 (1.52-1.82)**  **1.34 (1.23-1.47)**  **1.16 (1.08-1.25)**  1.00 (ref)  **0.91 (0.85-0.98)**  **0.80 (0.73-0.88)**  **0.64 (0.54-0.75)** |
|  |  |  |  |  |  | stroke  (ICD-10 codes I60-I61, I63-I64) | 3,161 | ≤10 11  12  13  14  15 ≥16 | **1.27 (1.06-1.53)**  1.10 (0.97-1.24)  1.00 (0.89-1.11)  1.00 (ref)  1.04 (0.94-1.16)  1.10 (0.97-1.25)  **1.25(1.08-1.44)** | < 40 40-44 45-49 50-51 52-53 54-55  ≥ 56 | **1.98 (1.57-2.48)**  **1.49 (1.28-1.72)**  **1.18 (1.06-1.32)**  1.00 (ref)  **0.87 (0.77-0.99)**  **0.75 (0.65-0.87)**  **0.72 (0.60-0.86)** | < 30  30-32 33-35 36-38 39-41 42-44  ≥ 45 | **1.75 (1.52-2.01)**  **1.18 (1.02- 1.37)**  **1.14 (1.01-1.29)**  1.00 (ref)  **0.88 (0.78-0.99)**  **0.75 (0.64-0.87)**  **0.56 (0.43-0.74)** |

HR, hazard ratio; SBP, systolic blood pressure; BMI, body mass index; WC, waist circumstance; TC, total cholesterol; HDL, high-density lipoprotein cholesterol; LDL, low-density lipoprotein cholesterol; DM, diabetes mellitus; HTN, hypertension; DL, dyslipidemia; MI, myocardial infarction; HRT, hormone replacement therapy; OC, oral contraceptive

* Subgroup analysis data for post-menopausal women among the total population (pre- and post-menopausal women) are presented when available.

^a^ included in the models for age at menarche

^b^ included in the models for age at menopause

**Table S2** Information on missing data

| **Variables** | **Missing number** |
| --- | --- |
| Self-administered questionnaire on reproductive factors | |
| Age at menarche | 38,454 |
| Age at menopause | 130,708 |
| Parity | 6,469 |
| Duration of breast feeding | 14,502 |
| History of breast cancer | 9,349 |
| Oral contraceptive use | 105,276 |
| Hormone replacement therapy | 81,618 |
| Other factors | |
| Age | 8,758 |
| Smoking | 9,779 |
| Alcohol consumption | 30,325 |
| Physical activity | 21,784 |
| Height | 454 |
| Weight | 607 |
| Body mass index | 628 |
| Waist circumference | 671 |
| Fasting glucose | 495 |
| Systolic blood pressure | 703 |
| Diastolic blood pressure | 707 |
| Total cholesterol | 486 |
| High-density cholesterol | 690 |
| Low-density cholesterol | 716 |
| Triglyceride | 617 |
| Diabetes mellitus | 495 |
| Hypertension | 717 |
| Dyslipidemia | 486 |

**Table S3** Hazard ratios and 95% confidence intervals of myocardial infarction, ischemic stroke, and cardiovascular disease according to reproductive factors (age at menarche, age at menopause, and reproductive span) without lag periods.

| Reproductive factors | Subjects  (N) | Myocardial infarction | | | | Ischemic stroke | | | | Cardiovascular disease | | | |
| --- | --- | --- | --- | --- | --- | --- | --- | --- | --- | --- | --- | --- | --- |
|  |  | Events  (n) | IR | Model 1 | Model 2 | Events  (n) | IR | Model 1 | Model 2 | Events  (n) | IR | Model 1 | Model 2 |
| Age at menarche (years) | | | | | | | | | | | | | |
| ≤12 | 12,166 | 152 | 1.3 | 0.87  (0.74–1.02) | 0.87  (0.74–1.03) | 273 | 2.4 | **1.13**  **(1.00–1.28)** | **1.14**  **(1.01-1.29)** | 406 | 3.6 | 1.03  (0.93–1.14) | 1.03  (0.93–1.14) |
| 13–14 | 151,854 | 2,482 | 1.8 | 1  (Ref.) | 1  (Ref.) | 3,558 | 2.5 | 1  (Ref.) | 1  (Ref.) | 5,736 | 4.1 | 1  (Ref.) | 1  (Ref.) |
| 15 | 218,107 | 4,004 | 2.0 | 1.00  (0.95–1.05) | 0.99  (0.95–1.05) | 5,937 | 3.0 | 1.00  (0.96–1.05) | 1.00  (0.96-1.04) | 9,472 | 4.7 | 1.01  (0.98–1.04) | 1.00  (0.97–1.03) |
| 16 | 257,531 | 5,732 | 2.4 | **1.06**  **(1.01–1.11)** | **1.05**  **(1.00–1.10)** | 8,797 | 3.7 | **1.06**  **(1.02–1.11)** | **1.06**  **(1.01-1.10)** | 13,804 | 5.9 | **1.07**  **(1.04–1.10)** | **1.06**  **(1.03–1.09)** |
| ≥17 | 584,889 | 15,106 | 2.8 | **1.08**  **(1.03–1.12)** | **1.06**  **(1.02–1.11)** | 24,132 | 4.5 | **1.10**  **(1.06–1.14)** | **1.08**  **(1.05-1.12)** | 37,191 | 7.0 | **1.10**  **(1.07–1.13)** | **1.08**  **(1.05–1.11)** |
| Age at menopause (years) | | | | | | | | | | | | | |
| <40 | 20,938 | 754 | 3.9 | **1.42**  **(1.32–1.53)** | **1.41**  **(1.31–1.52)** | 1,144 | 6.0 | **1.31**  **(1.23–1.39)** | **1.30**  **(1.22–1.38)** | 1,782 | 9.5 | **1.35**  **(1.28–1.42)** | **1.34**  **(1.28–1.41)** |
| 40–45 | 123,969 | 3,663 | 3.2 | **1.24**  **(1.19–1.29)** | **1.24**  **(1.19–1.29)** | 5,791 | 5.1 | **1.19**  **(1.15–1.23)** | **1.18**  **(1.15–1.22)** | 8,922 | 8.0 | **1.21**  **(1.18–1.24)** | **1.21**  **(1.18–1.24)** |
| 46–50 | 556,691 | 12,889 | 2.5 | **1.13**  **(1.10–1.16)** | **1.13**  **(1.10–1.16)** | 20,110 | 3.9 | **1.11**  **(1.09–1.14)** | **1.11**  **(1.09–1.14)** | 31,303 | 6.2 | **1.12**  **(1.10–1.14)** | **1.12**  **(1.10–1.14)** |
| 51–54 | 453,147 | 8,502 | 2.0 | 1  (Ref.) | 1  (Ref.) | 12,974 | 3.1 | 1  (Ref.) | 1  (Ref.) | 20,480 | 4.9 | 1  (Ref.) | 1  (Ref.) |
| ≥55 | 69,802 | 1,668 | 2.6 | 1.00  (0.94–1.05) | 0.99  (0.94–1.05) | 2,678 | 4.2 | 1.01  (0.97–1.05) | 1.01  (0.97–1.05) | 4,122 | 6.5 | 1.00  (0.97–1.04) | 1.00  (0.97–1.03) |
| Reproductive span (years) * | | | | | | | | | | | | | |
| <30 | 167,907 | 5,181 | 3.4 | **1.33**  **(1.27-1.38)** | **1.32**  **(1.27–1.37)** | 8,227 | 5.4 | **1.27**  **(1.23–1.31)** | **1.26**  **(1.22–1.30)** | 12,641 | 8.4 | **1.29**  **(1.26–1.33)** | **1.28**  **(1.25–1.32)** |
| 30–33 | 371,432 | 9,011 | 2.6 | **1.20**  **(1.16-1.25)** | **1.20**  **(1.15–1.24)** | 14,137 | 4.1 | **1.17**  **(1.14–1.21)** | **1.16**  **(1.13–1.20)** | 21,963 | 6.5 | **1.19**  **(1.16–1.22)** | **1.18**  **(1.15–1.21)** |
| 34–36 | 401,857 | 8,111 | 2.2 | **1.13**  **(1.08-1.17)** | **1.12**  **(1.08–1.16)** | 12,351 | 3.3 | **1.10**  **(1.06–1.13)** | **1.09**  **(1.06–1.13)** | 19,456 | 5.3 | **1.11**  **(1.08–1.14)** | **1.10**  **(1.08–1.13)** |
| 37–40 | 240,950 | 4,202 | 1.9 | 1  (Ref.) | 1  (Ref.) | 6,401 | 2.9 | 1  (Ref.) | 1  (Ref.) | 10,123 | 4.6 | 1  (Ref.) | 1  (Ref.) |
| ≥41 | 42,401 | 971 | 2.5 | 1.03  (0.96-1.10) | 1.03  (0.96–1.10) | 1,581 | 4.1 | 1.05  (0.99–1.11) | 1.05  (1.00–1.11) | 2,426 | 6.3 | 1.04  (1.00–1.09) | 1.04  (1.00–1.09) |

IR, incidence rate per 1,000 person-years

Model 1: The full model included age and traditional cardiovascular risk factors (income, smoking, alcohol consumption, regular exercise, body mass index, systolic blood pressure, total cholesterol, fasting glucose, hypertension, diabetes mellitus, and dyslipidemia)

Model 2: The full model included age, traditional cardiovascular risk factors (income, smoking, alcohol consumption, regular exercise, body mass index, systolic blood pressure, total cholesterol, fasting glucose, hypertension, diabetes mellitus, and dyslipidemia), and reproductive factors (age at menarche, age at menopause, parity, duration of breast feeding, duration of hormone replacement therapy, and duration of oral contraceptive use)

*The full model included reproductive span instead of age at menarche and menopause in Model 2.

**Table S4** Hazard ratios and 95% confidence intervals of myocardial infarction, ischemic stroke, and cardiovascular disease according to reproductive factors (age at menarche, age at menopause, and reproductive span) adjustment for confounders based on directed acyclic graph.

| Reproductive factors | Subjects  (N) | Myocardial infarction | | | Ischemic stroke | | | Cardiovascular disease | | |
| --- | --- | --- | --- | --- | --- | --- | --- | --- | --- | --- |
|  |  | Events  (n) | IR | Model | Events  (n) | IR | Model | Events  (n) | IR | Model |
| Age at menarche (years) |  |  |  |  |  | |  |  |  |  |
| ≤12 | 12166 | 137 | 1.4 | **0.75**  **(0.63-0.89)** | 252 | 2.5 | **0.97**  **(0.85-1.10)** | 373 | 3.7 | **0.88**  **(0.80-0.98)** |
| 13–14 | 151854 | 2267 | 1.8 | 1(Ref.) | 3238 | 2.6 | 1(Ref.) | 5251 | 4.2 | 1(Ref.) |
| 15 | 218107 | 3712 | 2.1 | **1.14**  **(1.08-1.20)** | 5418 | 3.0 | **1.17**  **(1.12-1.22)** | 8718 | 4.9 | **1.16**  **(1.12-1.20)** |
| 16 | 257531 | 5268 | 2.5 | **1.37**  **(1.31-1.44)** | 8074 | 3.8 | **1.48**  **(1.42-1.54)** | 12700 | 6.1 | **1.44**  **(1.39-1.48)** |
| ≥17 | 584889 | 13797 | 2.9 | **1.58**  **(1.51-1.66)** | 22014 | 4.6 | **1.78**  **(1.72-1.85)** | 34025 | 7.2 | **1.70**  **(1.65-1.75)** |
| Age at menopause (years) |  |  |  |  |  | |  |  |  |  |
| <40 | 20,938 | 686 | 4.0 | **1.40**  **(1.30-1.52)** | 1048 | 6.2 | **1.31**  **(1.23-1.39)** | 1637 | 9.8 | **1.35**  **(1.28-1.42)** |
| 40–45 | 123,969 | 3372 | 3.3 | **1.24**  **(1.19-1.29)** | 5246 | 5.2 | **1.18**  **(1.14-1.22)** | 8154 | 8.2 | **1.20**  **(1.17-1.23)** |
| 46–50 | 556,691 | 11771 | 2.6 | **1.12**  **(1.09-1.15)** | 18400 | 4.1 | **1.11**  **(1.08-1.14)** | 28694 | 6.4 | **1.11**  **(1.09-1.14)** |
| 51–54 | 453,147 | 7833 | 2.1 | 1(Ref.) | 11860 | 3.2 | 1(Ref.) | 18815 | 5.1 | 1(Ref.) |
| ≥55 | 69,802 | 1519 | 2.6 | 1.00  (0.94-1.05) | 2442 | 4.3 | 1.03  (0.98-1.07) | 3767 | 6.7 | 1.01  (0.98-1.05) |
| Reproductive span (years) * |  |  |  |  |  | |  |  |  |  |
| <30 | 167,907 | 4735 | 3.5 | **1.16**  **(1.07-1.27)** | 7495 | 5.5 | **1.14**  **(1.06-1.22)** | 11567 | 8.6 | **1.14**  **(1.08-1.21)** |
| 30–33 | 371,432 | 8250 | 2.7 | **1.14**  **(1.07-1.21)** | 12909 | 4.3 | **1.09**  **(1.03-1.14)** | 20109 | 6.7 | **1.10**  **(1.06-1.15)** |
| 34–36 | 401,857 | 7444 | 2.2 | **1.09**  **(1.04-1.14)** | 11298 | 3.4 | **1.06**  **(1.02-1.10)** | 17877 | 5.5 | **1.07**  **(1.04-1.10)** |
| 37–40 | 240,950 | 3861 | 1.9 | 1(Ref.) | 5842 | 2.9 | 1(Ref.) | 9284 | 4.7 | 1(Ref.) |
| ≥41 | 42,401 | 891 | 2.6 | 1.02  (0.94-1.12) | 1452 | 4.2 | 1.06  (0.99-1.13) | 2230 | 6.5 | 1.05  (0.99-1.11) |

IR, incidence rate per 1,000 person-years

For age at menarche, model was non-adjusted

For age at menopause, multivariable model included age, income, smoking, alcohol consumption, regular exercise, body mass index, age at menarche, parity, duration of breast feeding, oral contraceptives

For reproductive span, multivariable model included age, income, smoking, alcohol consumption, regular exercise, body mass index, age at menarche, age at menopause, parity, duration of breast feeding, oral contraceptives

**Table S5** Age at menarche in combination with age at menopause and reproductive span and the risk of cardiovascular events.

|  | | Age at menarche (years) | | | | |
| --- | --- | --- | --- | --- | --- | --- |
|  |  | ≤12 | 13–14 | 15 | 16 | ≥ 17 |
| **Myocardial Infarction** | | | | | | |
| Age at menopause | < 40 | 0.88 (0.28–2.73) | **1.35 (1.01–1.79)** | **1.69 (1.34–2.13)** | **1.44 (1.17–1.76)** | **1.50 (1.33–1.70)** |
|  | 40–45 | 1.18 (0.76–1.84) | **1.26 (1.10–1.44)** | **1.34 (1.20–1.51)** | **1.33 (1.19–1.48)** | **1.32 (1.21–1.44)** |
|  | 46–50 | 1.09 (0.85–1.39) | **1.13 (1.03–1.24)** | **1.13 (1.04–1.23)** | **1.22 (1.13–1.32)** | **1.21 (1.12–1.30)** |
|  | 51–54 | 0.77 (0.57–1.06) | 1 (Ref.) | 1.00 (0.91–1.09) | 1.07 (0.98–1.16) | **1.09 (1.01–1.18)** |
|  | ≥ 55 | 0.34 (0.11–1.05) | 1.01 (0.84–1.22) | 1.06 (0.90–1.24) | 0.98 (0.86–1.13) | 1.08 (0.98–1.19) |
| Reproductive span | < 30 | 0.58 (0.22–1.54) | 1.18 (1.00–1.39) | **1.34 (1.17–1.54)** | **1.31 (1.18–1.45)** | **1.29 (1.19–1.40)** |
|  | 30–33 | 1.33 (0.81–2.18) | **1.21 (1.06–1.38)** | **1.23 (1.11–1.36)** | **1.19 (1.09–1.31)** | **1.16 (1.07–1.25)** |
|  | 34–36 | 1.04 (0.68–1.59) | **1.12 (1.01–1.24)** | 1.07 (0.98–1.17) | 1.14 (1.05–1.24) | 1.70 (0.99–1.16) |
|  | 37–40 | 0.93 (0.72–1.20) | 1 (Ref.) | 0.96 (0.87–1.06) | 0.98 (0.89–1.08) | 0.98 (0.89–1.07) |
|  | ≥ 41 | 0.74 (0.52–1.06) | 0.90 (0.78–1.04) | 1.03(0.876–1.211) | 0.98 (0.83–1.16) | 1.17 (1.02–1.35) |
| **Ischemic stroke** | | | | | | |
| Age at menopause | < 40 | 1.24 (0.56–2.77) | **1.28 (1.01–1.63)** | **1.42 (1.16–1.74)** | **1.52 (1.30–1.79)** | **1.38 (1.25–1.52)** |
|  | 40–45 | **1.65 (1.20–2.28)** | **1.17 (1.05–1.31)** | **1.22 (1.11–1.35)** | **1.23 (1.12–1.34)** | **1.28 (1.20–1.38)** |
|  | 46–50 | 1.13 (0.92–1.39) | **1.10 (1.02–1.19)** | **1.12 (1.05–1.20)** | **1.19 (1.11–1.27)** | **1.22 (1.14–1.30)** |
|  | 51–54 | 1.17 (0.94–1.45) | 1 (Ref.) | 0.95 (0.88–1.03) | **1.08 (1.00–1.16)** | **1.10 (1.03–1.17)** |
|  | ≥ 55 | 1.44 (0.91–2.26) | 0.98 (0.84–1.15) | 1.01 (0.89–1.16) | 1.05 (0.92–1.17) | **1.11 (1.03–1.20)** |
| Reproductive span | < 30 | **2.00 (1.27–3.14)** | **1.18 (1.03–1.35)** | **1.28 (1.15–1.44)** | **1.24 (1.14–1.35)** | **1.27 (1.19–1.35)** |
|  | 30–33 | 1.15 (0.73–1.81) | 1.10 (0.98–1.23) | 1.09 (1.00–1.19) | **1.16 (1.07–1.25)** | **1.18 (1.11–1.25)** |
|  | 34–36 | 1.15 (0.81–1.63) | **1.09 (1.00–1.18)** | **1.10 (1.02–1.18)** | **1.13 (1.06–1.21)** | 1.06 (0.99–1.13) |
|  | 37–40 | 1.18 (0.97–1.43) | 1 (Ref.) | **0.91 (0.84–0.99)** | 1.02 (0.94–1.10) | 1.03 (0.96–1.11) |
|  | ≥ 41 | 1.15 (0.91–1.47) | 0.95 (0.84–1.07) | 0.99 (0.87–1.13) | 1.06 (0.93–1.21) | **1.21 (1.08–1.35)** |
| **Cardiovascular disease** | | | | | | |
| Age at menopause | < 40 | 1.01 (0.51–2.03) | **1.32 (1.09–1.59)** | **1.52 (1.30–1.77)** | **1.50 (1.32–1.71)** | **1.43 (1.33–1.54)** |
|  | 40–45 | **1.50 (1.16–1.95)** | **1.19 (1.09–1.30)** | **1.27 (1.18–1.37)** | **1.26 (1.17–1.35)** | **1.30 (1.24–1.38)** |
|  | 46–50 | 1.11 (0.94–1.30) | **1.11 (1.04–1.18)** | **1.13 (1.07–1.19)** | **1.21 (1.14–1.27)** | **1.22 (1.16–1.28)** |
|  | 51–54 | 1.00 (0.84–1.20) | 1 (Ref.) | 0.97 (0.92–1.03) | **1.08 (1.01–1.14)** | **1.09 (1.04–1.15)** |
|  | ≥ 55 | 0.99 (0.65–1.53) | 0.99 (0.88–1.12) | 1.03 (0.92–1.14) | 1.02 (0.93–1.11) | **1.10 (1.03–1.17)** |
| Reproductive span | < 30 | 1.41 (0.93–2.15) | **1.17 (1.05–1.30)** | **1.31 (1.20–1.43)** | **1.26 (1.18–1.35)** | **1.28 (1.22–1.35)** |
|  | 30–33 | 1.28 (0.92–1.79) | **1.14 (1.04–1.24)** | **1.15 (1.07–1.23)** | **1.18 (1.11–1.25)** | **1.17 (1.12–1.23)** |
|  | 34–36 | 1.11 (0.84–1.46) | **1.11 (1.04–1.18)** | **1.09 (1.03–1.15)** | **1.15 (1.09–1.21)** | **1.06 (1.01–1.12)** |
|  | 37–40 | 1.06 (0.90–1.24) | 1 (Ref.) | **0.94 (0.88–1.00)** | 1.00 (0.94–1.06) | 1.01 (0.95–1.071) |
|  | ≥ 41 | 1.00 (0.82–1.23) | 0.93 (0.85–1.02) | 1.01 (0.91 –1.12) | 1.03 (0.93–1.15) | **1.20 (1.10–1.31)** |

Data are expressed as adjusted hazard ratios (95% confidence intervals).

Hazard ratios were adjusted for age, cardiovascular risk factors (income, smoking, alcohol consumption, regular exercise, body mass index, systolic blood pressure, total cholesterol, fasting glucose, hypertension, diabetes mellitus, and dyslipidemia), and reproductive factors (parity, duration of breast feeding, duration of HRT, and duration of OC use)

**Table S6** Hazard ratios and 95% confidence intervals of cardiovascular disease by reproductive factors according to age group.

| Age group | Reproductive factors | Subject  (N) | Myocardial infarction | | | Ischemic stroke | | | Cardiovascular disease | | |
| --- | --- | --- | --- | --- | --- | --- | --- | --- | --- | --- | --- |
|  |  |  | Event  (n) | IR | HR (95% CI) | Event  (n) | IR | HR (95% CI) | Event  (n) | IR | HR (95% CI) |
| <60 years | Age at menarche (years) |  |  |  |  |  |  |  |  |  |  |
|  | ≤12 | 8,783 | 63 | 0.9 | 0.84 (0.65–1.09) | 78 | 1.1 | 0.90 (0.71–1.13) | 137 | 1.9 | 0.87 (0.73–1.04) |
|  | 13–14 | 97,093 | 853 | 1.1 | 1 (Ref.) | 1,008 | 1.3 | 1 (Ref.) | 1,802 | 2.2 | 1 (Ref.) |
|  | 15 | 123,360 | 1,134 | 1.1 | 1.02 (0.93–1.11) | 1,298 | 1.3 | 0.98 (0.90–1.06) | 2,365 | 2.3 | 1.00 (0.94–1.06) |
|  | 16 | 122,213 | 1,267 | 1.2 | 1.09 (1.00–1.19) | 1,522 | 1.5 | **1.10 (1.01–1.19)** | 2,707 | 2.7 | **1.10 (1.03–1.17)** |
|  | ≥17 | 214,874 | 2,464 | 1.4 | **1.14 (1.05–1.23)** | 2,947 | 1.6 | **1.13 (1.05–1.22)** | 5,252 | 3.0 | **1.14 (1.08–1.21)** |
|  | *P* for trend |  |  |  | <0.0001 |  |  | <0.0001 |  |  | <0.0001 |
|  | Age at menopause (years) |  |  |  |  |  |  |  |  |  |  |
|  | <40 | 6,872 | 102 | 1.8 | **1.73 (1.41–2.11)** | 97 | 1.7 | **1.44 (1.18–1.77)** | 192 | 3.4 | **1.57 (1.35–1.81)** |
|  | 40–45 | 47,661 | 534 | 1.3 | **1.33 (1.21–1.47)** | 592 | 1.5 | **1.29 (1.18–1.41)** | 1,079 | 2.7 | **1.29 (1.21–1.38)** |
|  | 46–50 | 265,236 | 2,657 | 1.2 | **1.13 (1.07–1.20)** | 3,099 | 1.4 | **1.12 (1.07–1.18)** | 5,591 | 2.5 | **1.13 (1.08–1.17)** |
|  | 51–54 | 231,937 | 2,316 | 1.2 | 1 (Ref.) | 2,828 | 1.5 | 1 (Ref.) | 5,005 | 2.6 | 1 (Ref.) |
|  | ≥55 | 14,617 | 172 | 1.4 | 0.95 (0.81–1.11) | 237 | 2.0 | 1.02 (0.90–1.17) | 396 | 3.3 | 0.99 (0.89–1.10) |
|  | *P* for trend |  |  |  | <0.0001 |  |  | <0.0001 |  |  | <0.0001 |
|  | Reproductive span (years) |  |  |  |  |  |  |  |  |  |  |
|  | <30 | 58,892 | 721 | 1.5 | **1.50 (1.36–1.65)** | 822 | 1.7 | **1.40 (1.28–1.53)** | 1,481 | 3.0 | **1.43 (1.34–1.53)** |
|  | 30–33 | 165,864 | 1,733 | 1.3 | **1.26 (1.17–1.36)** | 2,020 | 1.5 | **1.18 (1.10–1.26)** | 3,652 | 2.7 | **1.22 (1.16–1.28)** |
|  | 34–36 | 208,661 | 2,084 | 1.2 | **1.16 (1.08–1.25)** | 2,379 | 1.4 | 1.05 (0.98–1.12) | 4,336 | 2.5 | **1.10 (1.05–1.16)** |
|  | 37–40 | 122,352 | 1,131 | 1.1 | 1 (Ref.) | 1,470 | 1.4 | 1 (Ref.) | 2,526 | 2.5 | 1 (Ref.) |
|  | ≥41 | 10,554 | 112 | 1.3 | 1.00 (0.82–1.21) | 162 | 1.9 | 1.08 (0.92–1.27) | 268 | 3.1 | 1.05 (0.93–1.20) |
|  | *P* for trend |  |  |  | <0.0001 |  |  | <0.0001 |  |  | <0.0001 |
| ≥60 years | Age at menarche (years) |  |  |  |  |  |  |  |  |  |  |
|  | ≤12 | 3,383 | 74 | 2.7 | 0.87 (0.69–1.10) | 174 | 6.4 | **1.32 (1.13–1.54)** | 236 | 8.7 | **1.15 (1.01–1.31)** |
|  | 13–14 | 54,761 | 1,414 | 3.2 | 1 (Ref.) | 2,230 | 5.0 | 1 (Ref.) | 3,449 | 7.8 | 1 (Ref.) |
|  | 15 | 94,747 | 2,578 | 3.3 | 1.00 (0.94–1.07) | 4,120 | 5.4 | 1.00 (0.95–1.06) | 6,353 | 8.4 | 1.01 (0.97–1.05) |
|  | 16 | 135,318 | 4,001 | 3.6 | 1.04 (0.98–1.10) | 6,552 | 6.0 | **1.05 (1.00–1.11)** | 9,993 | 9.3 | **1.05 (1.01–1.09)** |
|  | ≥17 | 370,015 | 11,333 | 3.8 | 1.04 (0.98–1.09) | 19,067 | 6.4 | **1.07 (1.03–1.12)** | 28,773 | 9.8 | **1.06 (1.02–1.10)** |
|  | *P* for trend |  |  |  | 0.0674 |  |  | 0.0002 |  |  | <0.0001 |
|  | Age at menopause (years) |  |  |  |  |  |  |  |  |  |  |
|  | <40 | 14,066 | 584 | 5.2 | **1.33 (1.22–1.45)** | 951 | 8.5 | **1.27 (1.19–1.36)** | 1,445 | 13.1 | **1.30 (1.23–1.37)** |
|  | 40–45 | 76,308 | 2,838 | 4.6 | **1.20 (1.15–1.26)** | 4,654 | 7.7 | **1.15 (1.11–1.20)** | 7,075 | 11.8 | **1.17 (1.14–1.21)** |
|  | 46–50 | 291,455 | 9,114 | 3.9 | **1.11 (1.07–1.14)** | 15,301 | 6.5 | **1.11 (1.08–1.13)** | 23,103 | 10.0 | **1.11 (1.08–1.13)** |
|  | 51–54 | 221,210 | 5,517 | 3.0 | 1 (Ref.) | 9,032 | 5.0 | 1 (Ref.) | 13,810 | 7.8 | 1 (Ref.) |
|  | ≥55 | 55,185 | 1,347 | 3.0 | 0.99 (0.93–1.05) | 2,205 | 4.9 | 1.00 (0.95–1.05) | 3,371 | 7.6 | 0.99 (0.96–1.03) |
|  | *P* for trend |  |  |  | <0.0001 |  |  | <0.0001 |  |  | <0.0001 |
|  | Reproductive span (years) |  |  |  |  |  |  |  |  |  |  |
|  | <30 | 109,015 | 4,014 | 4.6 | **1.25 (1.19–1.31)** | 6,673 | 7.7 | **1.23 (1.19–1.28)** | 10,086 | 11.8 | **1.24 (1.20–1.28)** |
|  | 30–33 | 205,568 | 6,517 | 3.9 | **1.15 (1.10–1.20)** | 10,889 | 6.6 | **1.15 (1.11–1.19)** | 16,457 | 10.1 | **1.15 (1.12–1.19)** |
|  | 34–36 | 193,196 | 5,360 | 3.4 | **1.09 (1.04–1.14)** | 8,919 | 5.7 | **1.10 (1.07–1.15)** | 13,541 | 8.8 | **1.10 (1.07–1.13)** |
|  | 37–40 | 118,598 | 2,730 | 2.8 | 1 (Ref.) | 4,372 | 4.5 | 1 (Ref.) | 6,758 | 7.0 | 1 (Ref.) |
|  | ≥41 | 31,847 | 779 | 3.0 | 1.02 (0.94–1.11) | 1,290 | 5.0 | 1.06 (0.99–1.12) | 1,962 | 7.6 | 1.04 (0.99–1.09) |
|  | *P* for trend |  |  |  | <0.0001 |  |  | <0.0001 |  |  | <0.0001 |

IR, incidence rate per 1,000 person-years; HR, hazard ratio; CI, confidence interval

Age at menarche, Age at menopause: The full model included age, cardiovascular risk factors (income, smoking, alcohol consumption, regular exercise, body mass index, systolic blood pressure, total cholesterol, fasting glucose, hypertension, diabetes mellitus, and dyslipidemia), and reproductive factors (age at menarche, age at menopause, parity, duration of breast feeding, duration of hormone replacement therapy, and duration of oral contraceptive use)

Reproductive span: The full model included age, cardiovascular risk factors (income, smoking, alcohol consumption, regular exercise, body mass index, systolic blood pressure, total cholesterol, fasting glucose, hypertension, diabetes mellitus, and dyslipidemia), and reproductive factors (reproductive span, parity, duration of breast feeding, duration of hormone replacement therapy, and duration of oral contraceptive use)
