# Supplementary figures and images for: Associations of reproductive factors with incidence of myocardial infarction and ischemic stroke in postmenopausal women: a cohort study
Source: BMC Med. 2023 Feb 20;21:64. doi: 10.1186/s12916-023-02757-2 (PMC9942298; doi:10.1186/s12916-023-02757-2)

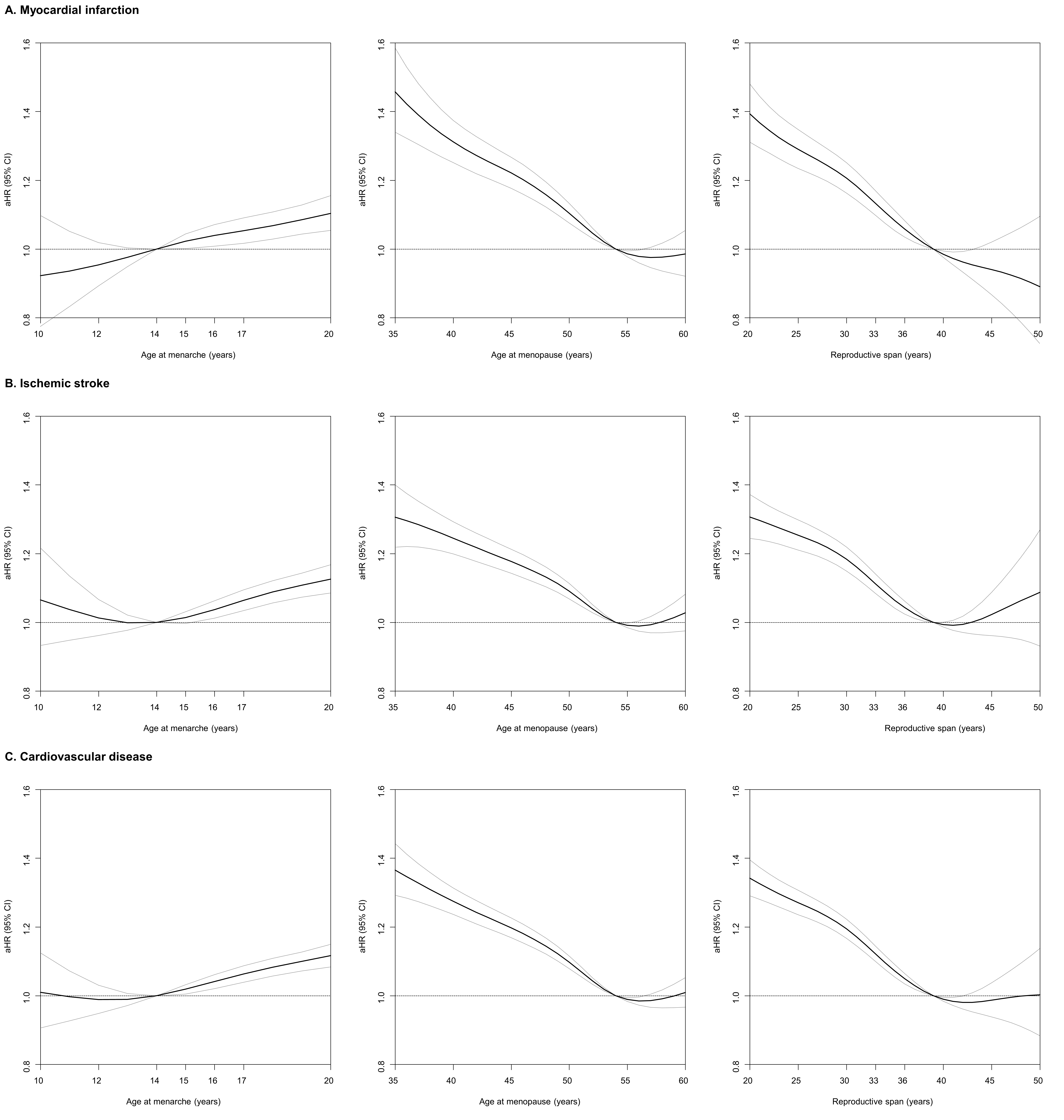

Supplement: Supplementary file 2 — Additional file 2: Fig. S1. The restricted cubic spline curve for the association of reproductive factors with cardiovascular diseases. [file 12916_2023_2757_MOESM2_ESM.tif]

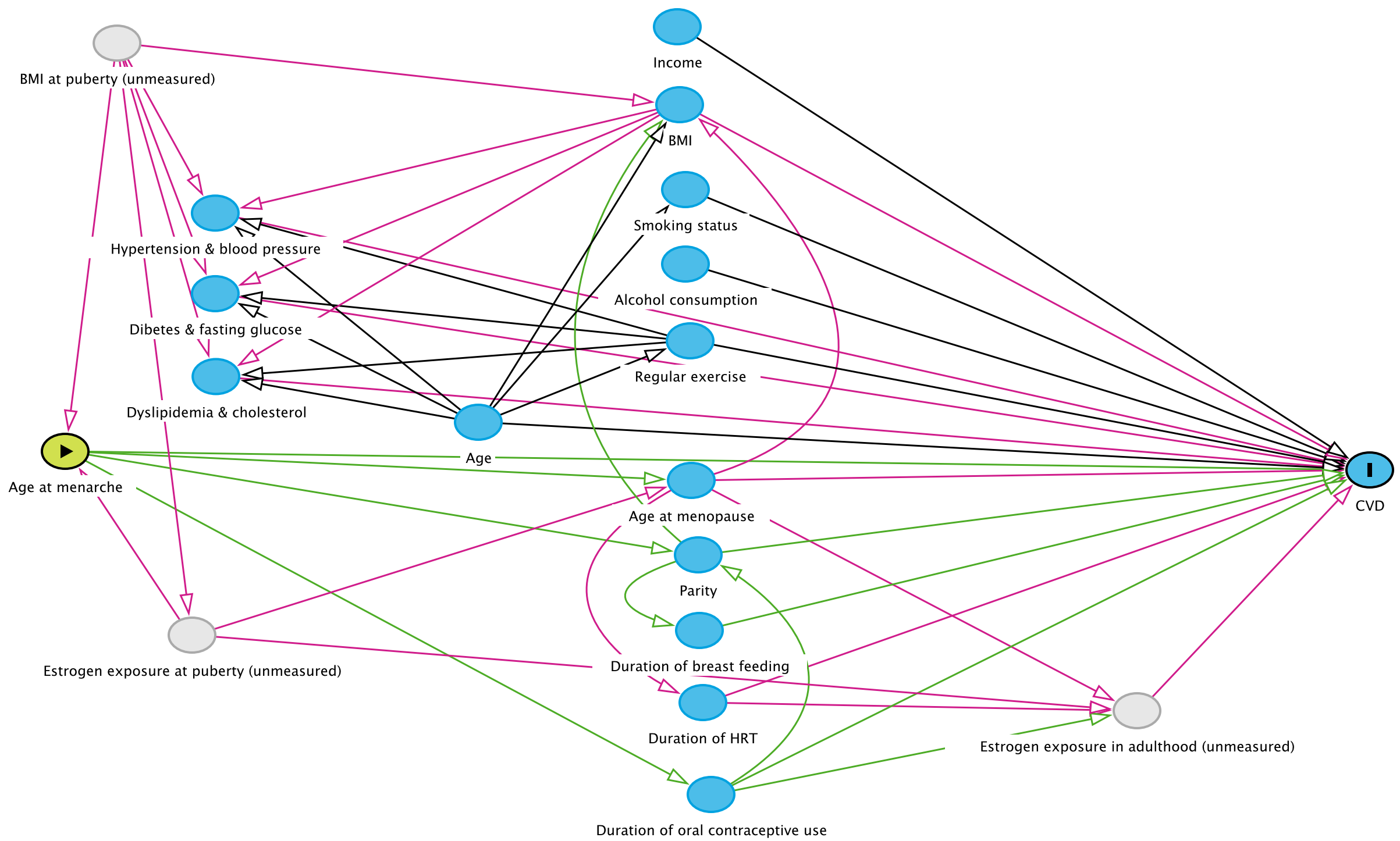

Supplement: Supplementary file 3 — Additional file 3: Fig. S2. Directed acyclic graph illustrating the assumptions about the causal relationship between age at menarche and cardiovascular disease. Red circles indicate confounders, blue circles represent mediators/colliders. The grey circle refers to an unmeasured variable. BMI, body mass index; HRT, hormone replacement therapy; CVD, cardiovascular disease [file 12916_2023_2757_MOESM3_ESM.pdf]

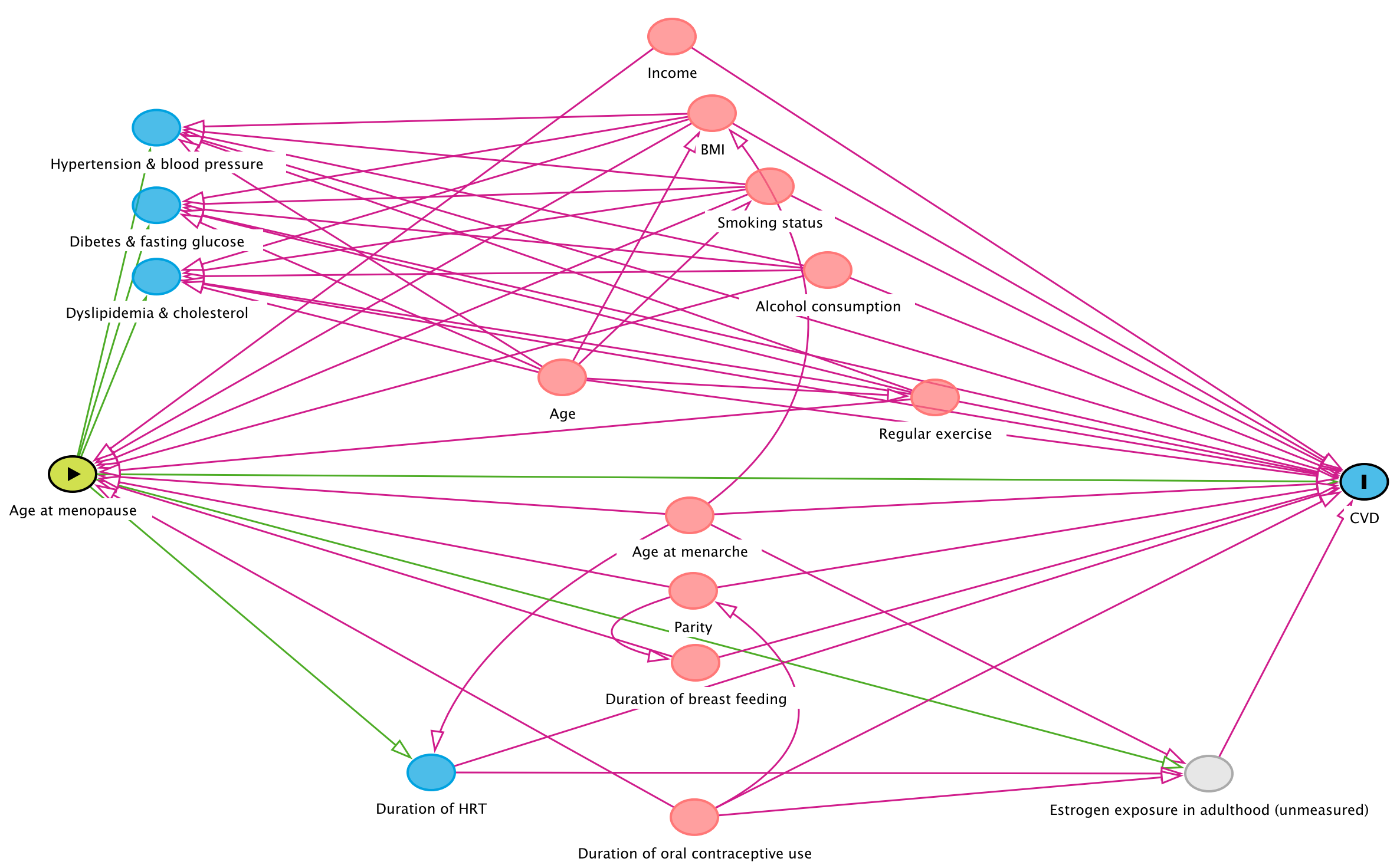

Supplement: Supplementary file 4 — Additional file 4: Fig. S3. Directed acyclic graph illustrating the assumptions about the causal relationship between age at menopause and cardiovascular disease. Red circles indicate confounders, blue circles represent mediators/colliders. The grey circle refers to an unmeasured variable. BMI, body mass index; HRT, hormone replacement therapy; CVD, cardiovascular disease. [file 12916_2023_2757_MOESM4_ESM.pdf]

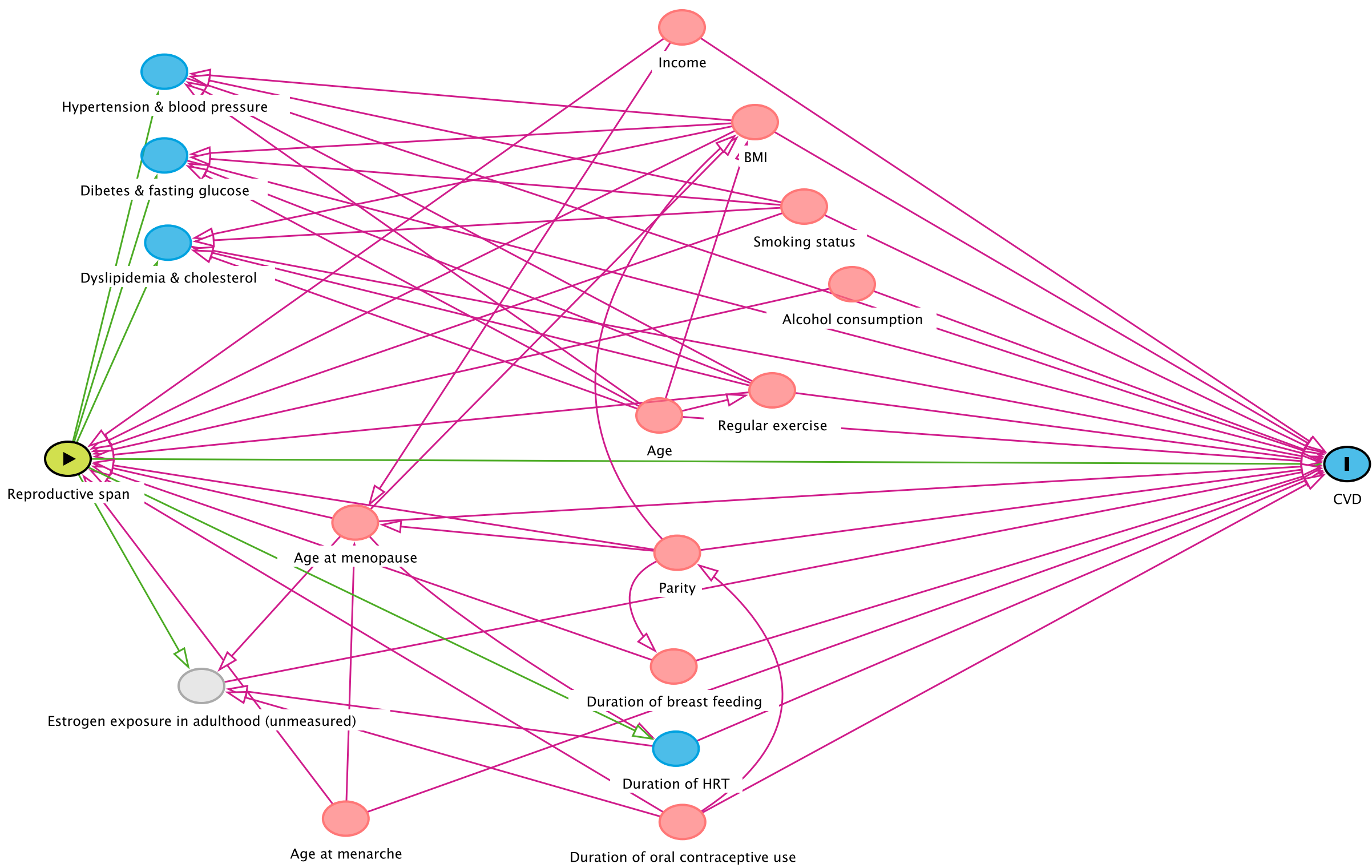

Supplement: Supplementary file 5 — Additional file 5: Fig. S4. Directed acyclic graph illustrating the assumptions about the causal relationship between reproductive span and cardiovascular disease. Red circles indicate confounders, blue circles represent mediators/colliders. The grey circle refers to an unmeasured variable. BMI, body mass index; HRT, hormone replacement therapy; CVD, cardiovascular disease. [file 12916_2023_2757_MOESM5_ESM.pdf]

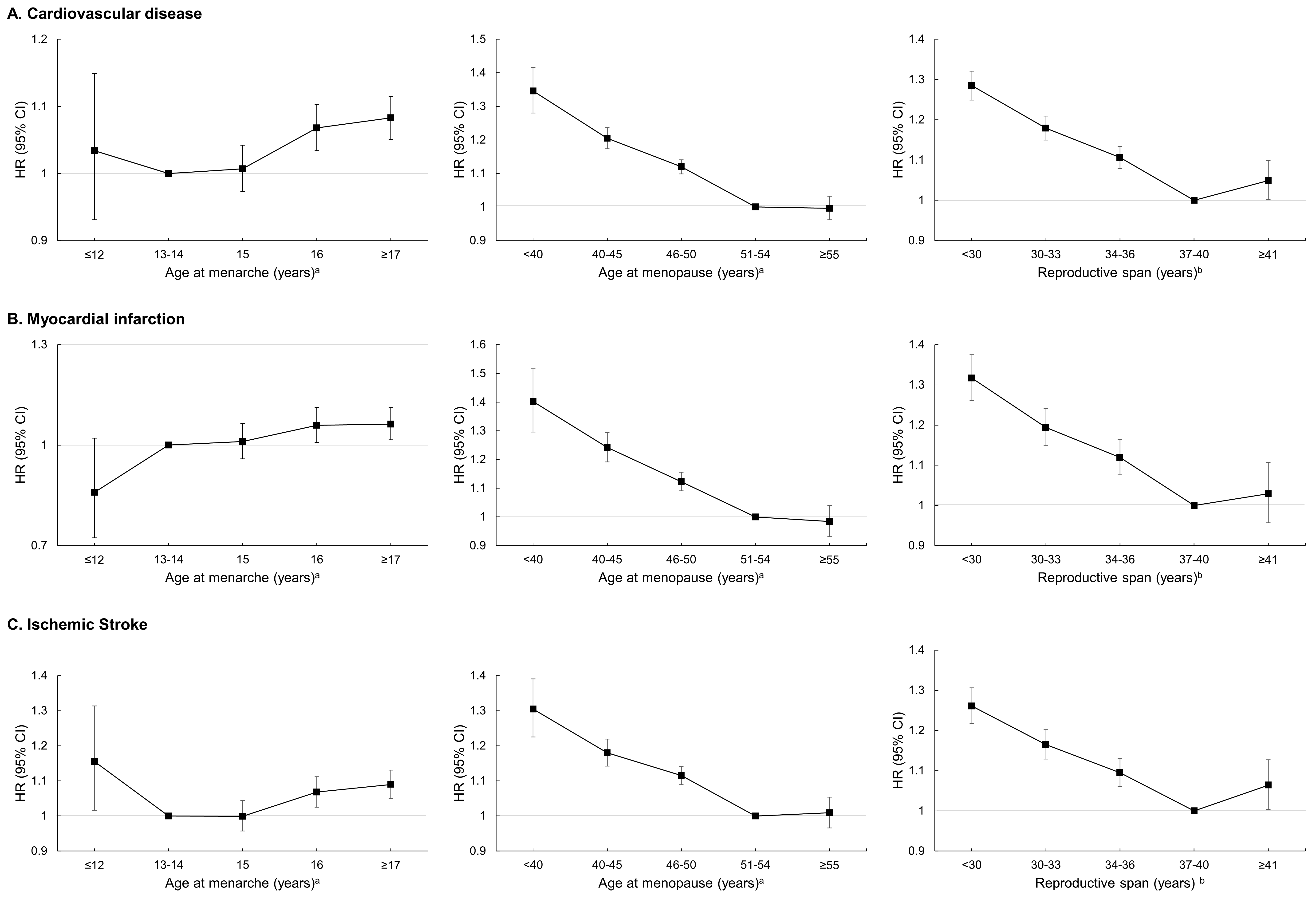

Supplement: Supplementary file 6 — Additional file 6: Fig. S5. Hazard ratios (HRs) and 95% confidence intervals (CIs) for cardiovascular events according to menstrual history. aModel 2: The full model included age, cardiovascular risk factors (income, smoking, alcohol consumption, regular exercise, body mass index, systolic blood pressure, total cholesterol, fasting glucose, hypertension, diabetes mellitus, and dyslipidemia) and reproductive factors (age at menarche, age at menopause, parity, duration of breast feeding, duration of hormone replacement therapy, and duration of oral contraceptive use). bModel 3: The full model included age, cardiovascular risk factors (income, smoking, alcohol consumption, regular exercise, body mass index, systolic blood pressure, total cholesterol, fasting glucose, hypertension, diabetes mellitus, and dyslipidemia) and reproductive factors (reproductive span, parity, duration of breast feeding, duration of hormone replacement therapy, and duration of oral contraceptive use) [file 12916_2023_2757_MOESM6_ESM.tif]
